# Effectiveness and Cost-Effectiveness of a Self-Guided Internet Intervention for Social Anxiety Symptoms in a General Population Sample: Randomized Controlled Trial

**DOI:** 10.2196/16804

**Published:** 2020-01-10

**Authors:** John Powell, Veronika Williams, Helen Atherton, Kylie Bennett, Yaling Yang, Mina Davoudianfar, Annika Hellsing, Angela Martin, Jill Mollison, Milensu Shanyinde, Ly-Mee Yu, Kathleen M Griffiths

**Affiliations:** 1 Nuffield Department of Primary Care Health Sciences University of Oxford Oxford United Kingdom; 2 School of Nursing Faculty of Education and Professional Studies Nipissing University North Bay, ON Canada; 3 Unit of Academic Primary Care Warwick Medical School University of Warwick Coventry United Kingdom; 4 eHub Health Pty Ltd Goulburn NSW Australia; 5 Research School of Psychology Australian National University Canberra Australia

**Keywords:** randomized controlled trial, internet, self-care, social anxiety

## Abstract

**Background:**

Many people are accessing digital self-help for mental health problems, often with little evidence of effectiveness. Social anxiety is one of the most common sources of mental distress in the population, and many people with symptoms do not seek help for what represents a significant public health problem.

**Objective:**

This study aimed to evaluate the effectiveness of a self-guided cognitive behavioral internet intervention for people with social anxiety symptoms in the general population.

**Methods:**

We conducted a two-group randomized controlled trial in England between May 11, 2016, and June 27, 2018. Adults with social anxiety symptoms who were not receiving treatment for social anxiety were recruited using online advertisements. All participants had unrestricted access to usual care and were randomized in a 1:1 ratio to either a Web-based unguided self-help intervention based on cognitive behavioral principles or a waiting list control group. All outcomes were collected through self-report online questionnaires. The primary outcome was the change in 17-item Social Phobia Inventory (SPIN-17) score from baseline to 6 weeks using a linear mixed-effect model that used data from all time points (6 weeks, 3 months, 6 months, and 12 months).

**Results:**

A total of 2122 participants were randomized, and 6 were excluded from analyses because they were ineligible. Of the 2116 eligible randomized participants (mean age 37 years; 80.24%, 1698/2116 women), 70.13% (1484/2116) had follow-up data available for analysis, and 56.95% (1205/2116) had data on the primary outcome, although attrition was higher in the intervention arm. At 6 weeks, the mean (95% CI) adjusted difference in change in SPIN-17 score in the intervention group compared with control was −1.94 (−3.13 to −0.75; *P*=.001), a standardized mean difference effect size of 0.2. The improvement was maintained at 12 months. Given the high dropout rate, sensitivity analyses explored missing data assumptions, with results that were consistent with those of the primary analysis. The economic evaluation demonstrated cost-effectiveness with a small health status benefit and a reduction in health service utilization.

**Conclusions:**

For people with social anxiety symptoms who are not receiving other forms of help, this study suggests that the use of an online self-help tool based on cognitive behavioral principles can provide a small improvement in social anxiety symptoms compared with no intervention, although dropout rates were high.

**Trial Registration:**

ClinicalTrials.gov NCT02451878; https://clinicaltrials.gov/ct2/show/NCT02451878

## Introduction

### Background

Many people are accessing digital tools for self-help for a range of mental health problems [1]. Social anxiety is one of the most common sources of mental distress in the population and represents a significant public health problem [2]. It is characterized by a cluster of cognitive, behavioral, and physiological symptoms including an intense and persistent fear of being negatively evaluated in social or performance situations, along with avoidance of such situations. The individual fears that they may act in such a way, or show anxiety symptoms, which would lead to embarrassment or humiliation. A diagnosis of social anxiety disorder may be made when symptoms are persistent and lead to disruption of daily routine, and work or social life, or if the symptoms themselves cause marked distress. There is a spectrum of symptomatology in the general population, and even subclinical symptoms that do not reach a clinical diagnostic threshold can cause substantial impairment [3,4].

Effective psychological and pharmacological treatments exist for social anxiety symptoms, but many people with symptoms do not seek or receive these treatments [5-7]. Self-guided digital tools have received much attention owing to their potential for high scalability and low marginal cost, in addition to the benefits of convenient access and anonymity they offer to people with social anxiety symptoms who may not seek help through more traditional routes because of embarrassment or fear of scrutiny [8]. A 2014 meta-analysis of randomized controlled trials of unguided internet-based self-help for social anxiety disorder identified 5 studies (270 participants in total) showing evidence for effectiveness for these interventions with a pooled standardized mean difference of 0.66 (95% CI 0.39-0.94) [5]. Subsequent trials using self-help interventions that use cognitive behavioral approaches have found similar effect sizes (between-group effect sizes ranging from 0.47 to 0.76) [9-14]. Previous studies were conducted on a relatively small scale (the largest number of participants in the intervention group in any previous individual study was 100 [14]) and have generally been confined to cases of social anxiety of clinical severity, usually based on a structured interview assessment. Very little work has attempted to examine the value of unguided self-help in a real-world context, where individuals self-select as requiring help with symptoms that may not reach a clinical threshold but may be causing them some level of distress and choose to access digital tools themselves, with no clinician contact at all. In this study, we examine the effectiveness and cost-effectiveness of the self-help E-couch social anxiety tool (described in detail below). This was chosen as it is a self-directed online intervention based on cognitive behavioral therapy principles including components of known effectiveness in face-to-face therapy. A previous laboratory-based comparative study of the E-couch social anxiety tool with 21 participants (mainly university students) in the E-couch arm showed pretest to posttest improvement in social anxiety measures [15].

### Objectives

We, therefore, undertook the first large-scale pragmatic randomized trial of an online self-guided cognitive behavioral intervention for people with self-reported social anxiety symptoms in the general population. Our experimental hypothesis was that participants who received the intervention would have a greater improvement in symptoms of social anxiety compared with participants who did not.

## Methods

### Trial Design and Participants

A two-arm, parallel-group randomized controlled trial was conducted to compare the effectiveness and cost-effectiveness of a Web-based and mobile-optimized self-guided intervention with a waiting list control condition for treating social anxiety symptoms. The study received ethics approval from the University of Oxford Medical Sciences Inter-Divisional Research Ethics Committee (MS-IDREC-C1-2015-167) and the Australian National University Human Research Ethics Committee (Protocol 2015/229) and is registered on ClinicalTrials.gov (NCT02451878). All participants provided informed consent to take part in the study using a self-completion online form. Outcomes were assessed at baseline, 6 weeks, 3 months, 6 months, and 12 months. The main follow-up time points were chosen to measure immediate effect (6 weeks as the intervention was designed to be used over a 6-week period) and long-term outcomes (12 months), along with interim time points (3 months and 6 months) to strengthen our repeated measures analysis and to support participant engagement. All study administration was conducted using automated online systems. The trial protocol is in Multimedia Appendix 1.

Participants were recruited primarily through an online advertisement placed on the UK National Health Service (NHS) website. In addition, study advertisements seeking individuals with social anxiety symptoms were placed on university and charity websites and disseminated via email and social media. We aimed to capture people with a broad range of social anxiety symptoms in the general population, who were likely to be typical of those seeking help from self-directed digital tools. Interested potential participants completed an online screening questionnaire to assess eligibility. We excluded anyone currently receiving therapist-guided treatment for social anxiety disorder or who self-reported a diagnosis of schizophrenia or bipolar affective disorder. Initial inclusion criteria were having access to the internet-based intervention, aged 18 years or older, resident in England, having an email address and mobile telephone number (to receive study emails and text alerts), and an initial criterion of scoring in a subclinical range of 13 to 19 on the 17-item Social Phobia Inventory (SPIN-17). We had initially chosen the 13 to 19 range with expert advice as this would, in theory, capture those scoring above the population mean score (11-12) while excluding those scoring above the commonly used threshold of 19, which indicates further assessment may be warranted (although this threshold does not represent a diagnosis). However, early in recruitment, it became apparent that most people in the general population volunteering for this study scored much higher than this, and the distribution of SPIN-17 scores meant that very few scored in the low range. There was clear evidence of a high level of unmet need among individuals living with social anxiety symptoms in the community and not seeking help elsewhere. With advice from our independent Trial Steering Committee, we, therefore, revised and reregistered the protocol (in line with good practice in clinical trials) to modify the inclusion criteria to include all individuals scoring 13 or more on SPIN-17, therefore capturing those in our hypothesized subclinical range of 13 to 19, as well as those with a higher score. We continued to exclude anyone receiving professional help, and therefore, the final sample represented adults in the general population who self-reported some level of social anxiety symptoms but who were not receiving treatment for social anxiety. Potentially eligible participants completed consent, and 24 hours later, they were sent an email link to self-complete their baseline measures using online questionnaires.

### Randomization and Masking

Once baseline measures had been completed, participants were randomized (1:1 ratio) to either the intervention group (E-couch) or the waiting list control group using a computer-generated random number sequence run through an automatic online program using a block size of 2 without stratification. Due to the nature of the intervention, participants were not blind to allocation.

### Interventions

Given that this was a general population sample, all participants continued to receive usual care. Participants in the intervention arm were given access to a password-protected website that contained the E-couch social anxiety module. The website was mobile-optimized and could, therefore, be used on a smartphone with the look and feel of a dedicated app, or on a computer browser.

#### Self-Guided Intervention

The E-couch social anxiety module is a self-directed interactive program based on cognitive behavioral therapy principles. The program is divided into 6 modules: a literacy section, which provides information about the symptoms of social anxiety, types of available help, and effective treatments, and 5 toolkits comprising exposure practice, cognitive restructuring (modifying your thinking), attention practice, social skills training, and relaxation. The content of the toolkits consists of evidence-based information, interactive exercises, and workbooks based on cognitive behavioral principles; participants could complete the modules in any order. Participants were advised to access and use the intervention over the initial period of 6 weeks (although they could work through the intervention at their own pace and were able to access it for the full 12-month duration of the study). “Ideal” usage of the intervention would entail engagement with 1 new module each week and ongoing updates to diaries and workbooks based on the user’s real-life experiences. E-couch was developed by the ehub team at the Australian National University National Institute for Mental Health Research. The intervention was adapted for this study to create a “stand-alone” social anxiety intervention that was accessed via a password-protected portal and with the usual E-couch branding removed. The program was adapted for a UK audience by removing Australian-specific terminology and undertaking user testing on the new version. No changes were made to the intervention during the study period.

#### Waiting List Control

Participants in the control group were informed that they had been put on a waiting list to receive access to the intervention in 12 months. They were asked to complete baseline and follow-up measures at the same time as participants in the intervention group. They received no other intervention.

Automated text (SMS) message and email reminders were sent to participants in both groups to reduce attrition. Participants in the intervention condition received 1 text message within 24 hours of randomization to remind them to access the intervention and 3 email reminders during the 6-week intervention period to remind them to log in to access the program. In addition, all participants received email invitations to complete follow-up surveys at each outcome measure time point, with those who failed to complete receiving a reminder email followed by a reminder SMS text message.

### Outcomes

The primary outcome was the change in SPIN-17 score from baseline to 6 weeks. The SPIN-17 is a 17-item self-rated scale covering the main social anxiety symptoms of fear, avoidance, and physiological disturbance. The responses to 17 statements (such as “I avoid talking to people I don’t know”) are rated on a 5-point scale from “not at all” (score=0) to “extremely” (score=4) to indicate the extent to which each statement reflects how the respondent was feeling in the past week, with higher scores reflecting greater social anxiety symptoms. The SPIN-17 has good test-retest reliability, internal consistency, and convergent and divergent validity [16]. Secondary outcomes were all also self-report measures with good reliability and validity: the 8-item Brief Fear of Negative Evaluation (BFNE-S scale), which is very commonly used in studies of social anxiety and measures one of the key psychological constructs of social anxiety (example item: “I am frequently afraid of other people noticing my shortcomings”) [17]; the 20-item Centre for Epidemiologic Studies Depression scale (CES-D), which has been widely used in online studies of anxiety and depression interventions to measure depressive symptoms [18]; the 7-item Short Warwick-Edinburgh Mental Well-Being Scale (SWEMWBS), a measure of mental well-being requiring participants to provide the extent of their agreement with statements about thoughts and feelings over the previous 2 weeks, which has been shown to be responsive to change (example item: “I’ve been feeling useful”) [19,20]; and the widely used and validated 36-item Short Form Health Survey (version 1) to measure health status and quality of life expressed in mental and physical component scores [21]. We also measured usage of the intervention in terms of number of E-couch modules completed, total time in minutes spent on modules, and total page views. Adverse events were not anticipated, but participants were asked to self-report any ill effects thought to be related to the intervention.

### Sample Size

We aimed to recruit 2104 participants (ie, 1052 per group) to this trial, to detect a small between-group standardized mean difference of 0.2 at 5% two-sided significance level and 90% power, assuming a high level of potential attrition of up to 50% given the fully self-guided nature of the intervention and automated nature of the trial (all trial procedures were conducted online). Although previous studies have suggested a larger treatment effect for internet-delivered interventions, we believed this treatment effect is too optimistic for a pragmatic trial of a self-guided treatment in a general population sample. The target effect size, although small at an individual level, can potentially translate into an important population-level change [22].

### Statistical Analyses

The statistical analysis was finalized before unblinding of the data. Primary analysis was modified intention to treat according to allocated group irrespective of adherence and with at least one outcome questionnaire completed post randomization. A linear mixed-effect model was fitted to the primary outcome data, using data collected at 6 weeks, 3 months, 6 months, and 12 months. Participant was included as a random intercept. Randomized group, baseline SPIN-17 score, time, and time by randomized group interaction term were fitted as fixed effects. An unstructured variance covariance matrix was specified between repeated measures on the same individual. Assumptions of normality and constant variance for linear mixed-effects models were assessed by residual plots and other diagnostics plots.

Given the high level of attrition expected in online trials of self-guided digital interventions, we also prespecified a Complier Average Causal Effect (CACE) analysis for the primary outcome and 2 other main outcomes, to include only participants who completed at least one module of the intervention and at least one outcome assessment to investigate the effect of the intervention in participants who adhered to the intervention. An instrumental variable approach was adopted to provide the CACE estimate at 6 weeks. This method involved a 2 least squares (using the “ivregress 2sls” command in STATA SE Version 15.1, StataCorp, Texas) estimation from fitting a linear regression model of the primary outcome, adjusting for baseline SPIN-17 and compliance instrumented on randomized group [23].

Similar approaches were undertaken for other outcomes. A CACE analysis at 6 weeks was conducted on fear of negative evaluation (BFNE-S) and mental well-being (SWEMWBS) measures, similarly adjusting for baseline BFNE-S and SWEMWBS in the linear regression models. Safety analyses were not conducted as there were no adverse events reported during the study period.

In anticipation of high levels of dropout, we prespecified various sensitivity analyses to explore the impact of assumptions regarding missing data in the primary outcome analysis. These included analyses (1) of participants with complete data at all time periods, (2) adjusting for factors found to be predictive of missingness, (3) fitting a pattern mixture model to assess different degrees of missing not at random, as well as (4) an assessment of missing not at random assumption for the primary outcome by assuming plausible arm-specific differences of missing SPIN-17 score between responders (with SPIN-17 score at 6 weeks) and nonresponders (missing SPIN-17 score at 6 weeks) [24,25].

Predefined subgroup analyses were conducted on change at 6 weeks for SPIN-17, BFNE-S, CES-D, and SWEMWBS for baseline SPIN-17 (<19, ≥19) to ascertain if the benefit differed between groups scoring above or below the screening threshold and for certain demographic characteristics to determine if the effectiveness of the intervention differed by the individual characteristics we had measured, that is, age (≤35, >35 years), gender (male, female), educational level (degree, no degree), and ethnicity (any white, nonwhite). Subgroup analyses were conducted by inclusion of an interaction term of baseline subgroup by randomized group by time in the linear mixed model. Descriptive statistics were used to describe usage data, adherence, and self-reports of other help received during the study period. All statistical analyses were performed using STATA SE version 15.1 [26].

### Economic Evaluation

A cost utility analysis from an NHS and social care perspective was conducted within this trial to assess the cost-effectiveness of the intervention. The total costs of developing, modifying, delivering, and maintaining the intervention were obtained, and the mean intervention cost was estimated for the participants recruited in the intervention group. Data on health care service utilization (for any reasons) were collected for all participants, including primary care consultations, hospital outpatient appointments, and hospital admissions. Unit costs for these health services were obtained from the Personal Social Services Research Unit (2016-2017) using national average costs [27]. Maximum follow-up was 1 year; therefore, no discounting was applied. The total and mean costs for the intervention and the waiting list control group were calculated. Effectiveness was measured in quality-adjusted life years (QALYs) using the under-the-curve approach by combining the duration of follow-up with the health status utilities at the start and end points. Health status was measured using the self-reported SF-36 measure at baseline, 6 weeks, 3 months, 6 months, and 12 months. The analysis examined short-term (6 weeks) and long-term (12 months) impact. Health status utilities were converted from SF-36 to SF-6D indices using the established UK-based utility algorithm obtained through the University of Sheffield Licensing [28]. The primary outcome was the incremental cost per QALY gained between the intervention group at 6 weeks and 12 months.

## Results

### Participant Characteristics and Trial Flow

**Figure 1:**
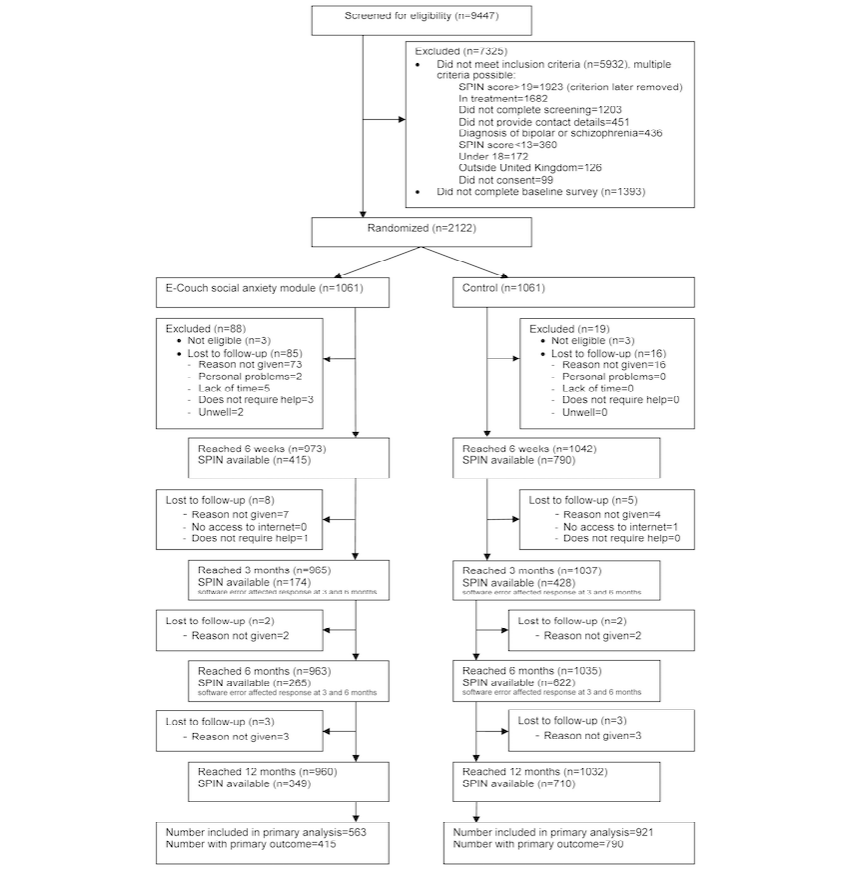
Consolidated Standards of Reporting Trials flow diagram.

Recruitment took place between May 11, 2016, and May 9, 2017, when the target sample size was reached. Participants were followed up for 1 year. Final data were locked on June 27, 2018 (allowing time for delayed 12-month follow-up responses). Figure 1 shows the flow diagram of the participants throughout the study period. We screened 9447 participants of whom 5932 (62.79%) were ineligible, and a further 1393 (14.74%) did not complete the baseline measures. We randomized 1061 (1061/2122, 50.00%) participants to E-couch and 1061 (1061/2122, 50.00%) to the control group. A total of 6 participants who were randomized to the study were excluded from all analyses because their later responses indicated they did not meet the inclusion criteria in terms of age, leaving 2116 participants randomized and included in analyses. Table 1 shows the baseline characteristics, which were similar across both groups. Owing to a software error, many participants were not sent the email requesting completion of their interim (3 months or 6 months) outcome measures. This error did not affect emails sent at the main follow-up time points of baseline, 6 weeks, and 12 months, and data from all time points were included in the analysis. Attrition rates differed significantly between groups with an overall loss to follow-up at the main follow-up time point (6 weeks) of 42.9%, with a loss of 60.8% in the intervention arm and 25.3% in the control arm (see Figure 1 and Multimedia Appendix 2). By 12 months, the primary outcome was available for 349 of 1061 (32.89%) participants in the intervention group and 710 of 1061 participants (66.92%) in the control group.

**Table 1:** Baseline characteristics of all participants randomized.

Baseline characteristics		E-couch (n=1058)	Control (n=1058)		Total randomized (N=2116)
Age (years), mean (SD); range		37.4 (13.9); 18-84	36.9 (13.6); 18-82	37.17 (13.8); 18-84	
**Gender, n (%)**					
	Female	859 (81.19)	839 (79.30)		1698 (80.25)
	Male	187 (17.67)	213 (20.13)		400 (18.90)
	Other	10 (0.95)	6 (0.57)		16 (0.76)
	Missing	2 (0.19)	0 (0.00)		2 (0.09)
**Marital status, n (%)**					
	Married or in a civil partnership	327 (30.91)	311 (29.40)		638 (30.15)
	Not married	727 (68.71)	745 (70.42)		1472 (69.57)
	Missing	4 (0.38)	2 (0.19)		6 (0.28)
**Education, n (%)**					
	Degree	514 (48.58)	538 (50.85)		1052 (49.72)
	No degree	544 (51.42)	520 (49.15)		1064 (50.28)
**Employment status, n (%)**					
	Employed	610 (57.66)	623 (58.88)		1233 (58.27)
	Unemployed	440 (41.59)	426 (40.26)		866 (40.93)
	Missing	8 (0.76)	9 (0.85)		17 (0.80)
**Income (£), n (%)**					
	≤25,000	736 (69.57)	739 (69.85)		1475 (69.71)
	>25,000	322 (30.43)	319 (30.15)		641 (30.29)
**Ethnicity, n (%)**					
	White	917 (86.67)	918 (86.77)		1835 (86.72)
	Nonwhite	141 (13.33)	140 (13.23)		281 (13.28)
Social Phobia Inventory-17, mean (SD); range		39.6 (13.1); 13-68	39.8 (13.4); 13-68		39.7 (13.3); 13-68
Mental well-being score (Short Warwick-Edinburgh Mental Well-Being Scale), mean (SD); range		17.7 (3.1); 7.0-30.7	17.7 (3.3); 7.0-35.0		17.7 (3.2); 7.0-35.0
Brief Fear of Negative Evaluation score, mean (SD); range		22.5 (7.1); 0-32	22.4 (7.3); 0-32		22.4 (7.2); 0-32
Centre for Epidemiologic Studies Depression scale, mean (SD); range		30.5 (12.3); 2-60	30.7 (12.2); 0-58		30.6 (12.2); 0-60
Short Form-36 (physical component summary), mean (SD); range		50.2 (10.3); 12.2-69.3	49.8 (10.6); 49.8 (10.6)		50.0 (10.4); 12.2-69.3
Short Form-36 (mental component summary), mean (SD); range		49.9 (9.5); 29.3-77.1	50.1 (9.4); 30.8-83.9		50.0 (9.5); 29.3-83.9

### Primary Outcome

Over the study period, there was a reduction of social anxiety symptoms in the E-couch group compared with that in the control group (see Multimedia Appendix 3). At 6 weeks, the E-couch group had a mean (SD) reduction of SPIN-17 score of −6.2 (10.8) and the control group −3.99 (9.3). The adjusted mean difference (95% CI; *P* value) in change in SPIN-17 score in E-couch compared with control was −1.94 (−3.13 to −0.75; *P*=.001; Table 2). This equates to a standardized mean difference effect size (between groups) of 0.2 (the pooled SD for SPIN-17 change was 9.81). At the 6-week follow-up, SPIN-17 outcome measures were available for 415 (415/1064, 39.00%) and 790 participants (790/1064, 74.25%) in the E-couch and control groups, respectively. In the CACE analysis, adjusted mean difference (95% CI; *P* value) in change in SPIN-17 score for intervention compared with control was −2.95 (−4.30 to −1.61; *P*<.001; Table 2). The results from the sensitivity analyses undertaken to explore missing data assumptions were also consistent with the primary outcome 6-week findings. These included analyses that only considered data from completers (defined as participants who returned all their outcome measures at the main time points of baseline, 6 weeks, and 12 months; see Multimedia Appendix 2) and the findings of the pattern mixture model even when assuming different missing data patterns in the intervention or control group. Finally, findings were also similar to the primary outcome analysis even under the assumption of missing not at random.

**Table 2:** Adjusted estimates from mixed-effect model for each outcome at 6 weeks and 12 months and estimates from the complier average causal effect analysis at 6 weeks.

Outcomes		Mixed-effect model analysis		Complier average causal effect analysis	
		Adjusted difference in mean change (95% CI)	*P* value	Adjusted difference in mean change (95% CI)	*P* value
**Social Phobia Inventory-17^a^**					
	E-couch vs control (6 weeks)	−1.94 (−3.13 to −0.75)	.001	−2.95 (−4.30 to −1.61)	<.001
	E-couch vs control (12 months)	−3.07 (−4.32 to −1.82)	<.001	N/A^b^	N/A
**Brief Fear of Negative Evaluation score**					
	E-couch vs control (6 weeks)	−1.09 (−1.79 to −0.38)	.003	−1.60 (−2.38 to −0.82)	<.001
	E-couch vs control (12 months)	−2.33 (−3.08 to −1.58)	<.001	N/A	N/A
**Short Warwick-Edinburgh Mental Well-Being Scale**					
	E-couch vs control (6 weeks)	0.38 (−0.02 to 0.77)	.06	0.59 (0.17 to 1.02)	.006
	E-couch vs control (12 months)	0.82 (0.39 to 1.24)	.001	N/A	N/A
**Centre for Epidemiologic Studies Depression scale**					
	E-couch vs control (6 weeks)	−3.35 (−4.54 to −2.15)	<.001	N/A	N/A
	E-couch vs control (12 months)	−1.79 (−3.06 to −0.52)	.006	N/A	N/A
**Short Form-36** **(physical component summary)**					
	E-couch vs control (6 weeks)	0.398 (−0.41 to 1.20)	.33	N/A	N/A
	E-couch vs control (12 months)	0.003 (−0.85 to 0.85)	.99	N/A	N/A
**Short Form-36** **(mental component summary)**					
	E-couch vs control (6 weeks)	1.06 (0.12 to 1.98)	.03	N/A	N/A
	E-couch vs control (12 months)	2.06 (1.07 to 3.06)	<.001	N/A	N/A

^a^Primary outcome.

^b^N/A: not applicable.

### Secondary Outcomes

Table 2 shows the results for the secondary outcomes. At the 12-month follow-up, participants randomized to the E-couch group continued to show a greater reduction in severity of social anxiety symptoms than the control participants, with a mean (95% CI; *P* value) adjusted difference in change in SPIN-17 score of −3.07 (−4.32 to −1.82; *P*<.001; Table 2). As with the primary outcome, the results of the sensitivity analyses exploring missing data assumptions were consistent with the main analysis SPIN-17 findings at 12 months (Multimedia Appendix 2). The findings for the other outcome measures of fear of negative evaluation (BFNE-S), mental well-being (SWEMWBS), depression (CES-D), and the mental component scale of the SF-36 all showed statistically significant small improvements favoring E-couch compared with control (see Table 2). There was no evidence of difference between groups for the physical component scale of the SF-36. All distributions of residuals from the fitted models satisfied the normality assumption. No adverse events were reported during the study period.

### Usage Data and Subgroup Analyses

At 6 weeks, the mean (SD) number of E-couch modules fully completed (out of 6) was 1.87 (1.43), total mean (SD) time in minutes spent on modules was 35.3 (48.1), and total mean (SD) page views was 37.6 (41.3). Greater adherence to the intervention was not associated with baseline SPIN-17 score, age, gender, or ethnicity (Multimedia Appendix 2). At 6 weeks, higher total page views or longer duration spent on modules was associated with larger improvement in social anxiety symptoms (Multimedia Appendix 2). At 6 weeks, E-couch had a significantly greater impact in improving social anxiety symptoms for participants with baseline SPIN-17 score greater than 19 (usually taken as cutoff to indicate clinical assessment warranted) compared with the few participants scoring in the lower range (SPIN-17 score 13-19; *P*=.01; Multimedia Appendix 2). In this subgroup analysis, the lower SPIN-17 scorers (the ones we had originally defined as a subclinical population) had no benefit from the intervention compared with the control group. The E-couch intervention also had a significantly greater beneficial impact on depressive symptoms at 6 weeks in participants with higher baseline SPIN-17 scores (*P*=.007), and again in this subgroup analysis, the few participants scoring in the lower SPIN-17 range had no benefit on depressive symptoms compared with the control group. There was no evidence of heterogeneity in the effects of intervention for the subgroup analyses involving BFNE-S and SWEMWBS.

### Economic Evaluation

At both 6-week and 12-month follow-ups, the waiting list control group, in general, used more health care services than the E-couch group (see Tables 3 and 4). This resulted in a mean health care cost saving of £26.48 at the 6-week follow-up and £65.04 at the 12-month follow-up. Adding the mean intervention cost of £48.40 to the intervention group, the E-couch group cost more than the control group at 6 weeks but is cost saving at 12 months. In the cost utility analysis, at both 6-week and 12-month follow-ups, there were very small improvements of general health status in both the E-couch group and the waiting list control group, with the E-couch group improvement slightly more than the control group: the SF-6D indices increased from 0.6 at baseline to 0.64 at 6 weeks and 0.66 at 12 months for the E-couch group and from 0.6 at baseline to 0.62 at 6 weeks and 0.64 at 12 months for the waiting list control group (see Table 5). At the 6-week follow-up, mean QALYs were 0.072 for the E-couch group and 0.070 for the control group, giving very small QALYs gains of 0.002 for the intervention over the control group. At the 12-month follow-up, mean QALYs were 0.635 for the E-couch group and 0.621 for the control group, with QALYs gain of 0.024 for the intervention over the control group. The incremental cost per QALY gained at 6 weeks was £10,960, which is highly likely to be cost-effective using accepted thresholds. At the 12-month follow-up, the E-couch dominated the waiting list control with more QALYs gained and less costs. Taking into consideration societal costs because of sick leave from work, the E-couch intervention was cost saving at both 6-week and 12-month follow-ups and, therefore, dominated the waiting list control.

**Table 3:** Health care utilization and other costs at 6 weeks (£).

Group	General practitioner attendance costs, mean (SD)	Outpatient attendance costs, mean (SD)	Inpatient costs, mean (SD)	Cost of work days lost to sick leave, mean (SD)	Mean health care cost (SD)	Mean societal cost (SD)
E-couch (n=383)	38 (89.52)	45.43 (123.99)	72.77 (461.03)	106.65 (476.99)	156.19 (527.42)	264.26 (764.82)
Waiting list (n=761)	38.55 (65.89)	42.49 (111.43)	101.64 (570.51)	123.78 (477.48)	182.67 (643.67)	308.38 (871.52)

**Table 4:** Health care utilization and other costs at 12 months (£).

Group	General practitioner attendance costs, mean (SD)	Outpatient attendance costs, mean (SD)	Inpatient costs, mean (SD)	Cost of work days lost to sick leave, mean (SD)	Mean health care cost (SD)	Mean societal cost (SD)
E-couch (n=324)	101.10 (152.66)	117.61 (319.44)	207.92 (864.18)	379.47 (1740.74)	425.30 (1077.82)	806.08 (2198.79)
Waiting list (n=680)	106.34 (198.02)	141.84 (416.68)	242.16 (1000.48)	375.18 (1248.74)	490.34 (1264.51)	869.43 (1823.07)

**Table 5:** Health status and quality-adjusted life years at baseline, 6 weeks and 12 months.

Group	Baseline	6 weeks		12 months	
	SF-6D^a^, mean (SD)	SF-6D, mean (SD)	QALY^b^, mean (SD)	SF-6D, mean (SD)	QALY, mean (SD)
E-couch	0.60 (0.10)^c^	0.64 (0.12)^d^	0.072 (0.012)^d^	0.66 (0.12)^e^	0.635 (0.10)^e^
Waiting list	0.60 (0.10)^c^	0.62 (0.11)^f^	0.070 (0.011)^f^	0.64 (0.12)^g^	0.621 (0.10)^g^

^a^SF-6D: six-dimensional health state short form.

^b^QALY: quality-adjusted life year.

^c^n=1061.

^d^n=377.

^e^n=324.

^f^n=753.

^g^n=675.

## Discussion

### Principal Findings

Our findings showed that this fully self-guided internet intervention gave a small reduction in social anxiety symptoms in participants recruited online from the general population, compared with a usual care waiting list control group, and this small but positive finding was robust to the sensitivity analyses, which explored our missing data assumptions. There was a similarly small but significant improvement in fear of negative evaluation. These improvements were also found in the CACE analyses and maintained at the 12-month follow-up. In the context of a very common mental health problem, this finding suggests that automated self-help delivered via the internet could reduce the overall level of social anxiety symptoms in the population, although at an individual level, the mean symptomatic benefit is small (*d*=0.2). The study findings provide no evidence as to whether this fully self-guided approach has a role in a clinical setting, where, to date, the evidence base suggests that although unguided self-help has effectiveness, therapist-guided and therapist-led approaches are likely to be superior. The cost-effectiveness analysis demonstrated that the intervention is likely to be cost-effective in both the short and long term, although the gain in general health status and QALY score was very small. The benefit seen in the condition-specific social anxiety outcome measures was greater than the general health status used in the cost utility analysis. Furthermore, the intervention cost could be substantially reduced if the E-couch is used by large numbers at a population level as a public health tool. Given that many people with social anxiety symptoms do not seek help, and that therapist-supported approaches are limited in supply, the findings suggest that unguided digital intervention for social anxiety can be beneficial for some people who do not access professional help and who are increasingly seeking support from apps and other digital tools. The self-help approach tested here might also complement face-to-face therapy, potentially reducing the amount of therapist contact time required and perhaps helping to maintain engagement, although these suggestions need to be empirically tested in future effectiveness and cost-effectiveness work.

### Comparison With Prior Work

This study adds to the body of work showing small positive effects for unguided digital self-help for social anxiety [5,9-14] and a range of other mental health problems [29]. Our effect size is smaller than that reported by others. Previous studies have had far fewer participants and usually required them to meet diagnostic criteria for social anxiety disorder. Our aim was to undertake a pragmatic trial addressing social anxiety symptoms (rather than disorder) among individuals in the general population. Our broad inclusion criteria, recruiting volunteers from the general population through internet adverts, including those with symptoms not reaching a diagnostic threshold, are likely to have contributed to the more modest benefit compared with previous work. We made the additional decision to conduct the trial in a fully automated and naturalistic way with no researcher contact to encourage intervention use. Our approach was intended to reflect the real-world situation of members of the public self-selecting digital tools and using them with no contact with health services.

### Strengths and Limitations

This study exemplifies both the strengths and weaknesses of undertaking online trials for digital interventions. We were able to recruit large numbers of participants from the general population using digital advertising, and we were able to deliver all measures and the intervention remotely, using little resources and with no requirement for any “real-world” contact between participants and researchers. The flipside of this was that, in common with other fully automated trials of unguided online interventions, there were high levels of dropout from the intervention and attrition from the trial [30]. This is commonly seen in internet research [31], including the higher level of retention in the control arm [32], which may be partly explained by these participants being on a waiting list and, therefore, having an incentive to keep returning to complete measures, and partly by participants in the intervention arm being required to “take action” (work through the intervention), whereas the control group could be more passive. Other possible reasons for dropout include some participants not liking the intervention, or feeling it was not working, or indeed dropping out because they felt they had improved and no longer needed it. The high loss to follow-up was compounded at the 3- and 6-month follow-up points by a software glitch, which reduced the number of emails sent to participants at this time. Fortunately, these were always intended as interim time points measured to contribute to the overall mixed linear model. We undertook sensitivity analyses and explored various approaches to adjusting for the missing data. All outcome measures were self-report with no observer-rated objective assessment. This was in line with our desire to deliver a fully automated trial, and the scales are well validated, but the subjective nature of these measures is a potential source of response bias. We did not employ a placebo but instead used a waiting list comparator whereby people received “usual care.” In other work, educational website placebos have often demonstrated an active effect [33]. Our pragmatic choice of control group, given that participants were not blind to allocation, may have introduced bias and increased the likelihood of a beneficial effect. Finally, most participants in this study were women. Social anxiety symptoms are twice as common in women than men [4], and women are more likely to seek health care generally [34]. Further work on the predictors and mediators of both adherence and response would be valuable [35,36].

### Conclusions

For people with social anxiety symptoms in the general population who are not receiving other forms of help, an online unguided tool based on cognitive behavioral principles accessed via a computer or mobile phone gave a small but significant improvement in social anxiety symptoms compared with no intervention. As with many online trials of digital interventions, we experienced a “methodological trade-off” between having a cheap, scalable model of intervention delivery versus the statistical challenge of a high degree of missing data. Our findings suggest this intervention could potentially offer the first self-help rung on the ladder of a stepped approach to social anxiety symptoms.

## Appendix

Multimedia Appendix 1 Study protocol.

Multimedia Appendix 2 Sensitivity, subgroup and additional analyses.

Multimedia Appendix 3 Descriptive statistics of Social Phobia Inventory (SPIN-17) score at all time points by randomized group.

Multimedia Appendix 4 CONSORT‐EHEALTH checklist (V 1.6.1).

## Abbreviations

ANU: Australian National University
BFNE-S: Brief Fear of Negative Evaluation
CACE: Complier Average Causal Effect
NHS: National Health Service
QALY: quality-adjusted life year
SPIN-17: 17-item Social Phobia Inventory
SWEMWBS: Short Warwick-Edinburgh Mental Well-Being Scale
