# Supplementary material for: Effectiveness and Cost-Effectiveness of a Self-Guided Internet Intervention for Social Anxiety Symptoms in a General Population Sample: Randomized Controlled Trial
Source: J Med Internet Res. 2020 Jan 10;22(1):e16804. doi: 10.2196/16804 (PMC6996778; doi:10.2196/16804)
Supplement: Multimedia Appendix 1 [file jmir_v22i1e16804_app1.docx]

**Version 5.0 Dated 13 October 2017**

**1. Title**

Effectiveness and cost-effectiveness of a fully self-guided internet-based intervention for sub-clinical social anxiety symptoms: pragmatic, population-based randomised controlled trial.

**2. Start date**

1^st^ June 2015

**3. Investigator team**

John Powell

Senior Clinical Researcher and Associate Professor

Nuffield Department of Primary Care Health Sciences

University of Oxford

*ROLE: Lead investigator, leads trial design and activity including fieldwork and analyses. Leads Oxford team. Responsible for delivery of whole project.*

Kathy Griffiths

Professor and Director

National Institute for Mental Health Research

Australian National University

*ROLE: Deputy lead investigator, leads ANU team, participates in the trial design, provides advice on consumer involvement and oversees the IT protocol development and trial activities at NIMHR, ANU.*

Ly-Mee Yu

Lead trial statistician

Nuffield Department of Primary Care Health Sciences

University of Oxford

*ROLE: Leads the statistical element, overall responsibility for all statistical analyses and will supervise statistician undertaking analyses.*

Yaling Yang

Senior Researcher in Health Economics

Nuffield Department of Primary Care Health Sciences

University of Oxford

*ROLE: Leads the health economics element, overall responsibility for all health economics analyses which she will undertake herself.*

Louise Locock

Associate Professor and Director of Applied Research, Health Experiences Research Group

Nuffield Department of Primary Care Health Sciences

University of Oxford

*ROLE: Leads the qualitative component. Will undertake some of the qualitative interviews and analyses and will also supervise the part-time Oxford research fellow (Helen Atherton) who will do some of this component.*

Kylie Bennett

Collaborative Research and Development Manager

National Institute for Mental Health Research

Australian National University

*ROLE: Refining the design and the development of the trial protocol for conversion into a technical specification, supervising the day-to-day work of the ANU trial manager.*

Anthony Bennett

IT Manager

National Institute for Mental Health Research

Australian National University

*ROLE: Responsible for designing and overseeing the development of the customised online research infrastructure underpinning the trial.*

Helen Atherton

Assistant Professor

Primary CareUniversity of Warwick

*ROLE: Postdoc research fellow working on project as a whole, alongside (and supervised by) the lead investigator. Will have leading role in producing all outputs such as journal papers and conference presentations.*

Mina Davoudianfar

Clinical Trial Manager

Nuffield Department of Primary Care Health Sciences

University of Oxford

*ROLE: Trial manager coordinating Oxford Primary Care Clinical Trials Unit contribution, responsible for ethics and governance clearances, and ensuring compliance with all CTU standard operating procedures (e.g. around consent, confidentiality, data management, quality assurance etc.).*

Veronika Williams

Senior Researcher

Nuffield Department of Primary Care Health Sciences

University of Oxford

*ROLE: Postdoc research fellow working on project as a whole, specifically leading the qualitative study, alongside (and supervised by) the lead investigator. Will have leading role in producing all outputs such as journal papers and conference presentations*

**4. Abstract**

*Design:*

A pragmatic, randomised controlled trial and economic evaluation to investigate the effectiveness and cost-effectiveness of the self-directed E-Couch social anxiety module versus a waiting list control condition, for reducing sub-clinical social anxiety symptoms in the general population.

*Study Population:*

Respondents (aged 16+) to a direct-to-consumer advertisement placed on national websites who have social anxiety symptoms but do not meet the criteria for social anxiety disorder (this latter group will instead be advised to seek help).

*Intervention and Control:*

Intervention is the E-Couch social anxiety module, a self-guided intervention. Control group receive no intervention and are placed on a waiting list to receive the intervention at the end of the trial. Both groups receive email and text message reminders.

*Outcome Measures:*

Change in self-reported social anxiety score using the Social Phobia Inventory (SPIN). Secondary measures: Brief Fear of Negative Evaluation scale (8 item BFNE-S); SOPHS Social Phobia screener; depression (CES-D); mental wellbeing (SWEMWEBS); quality of life (SF36); use of mental health services; safety events; adherence, retention and attrition rates. All measures at 6 weeks, 3, 6 and 12 months.

*Analysis:*

A mixed effects model will be used to analyse the primary outcome (intention to treat analysis).

*Sample Size:*

2104 participants (1051 per group) will provide 90% power to detect a small between group effect size of Cohen's d=0.2 at 5% two-sided significant level, accounting for a high level of potential attrition of up to 50%.

*Economic Evaluation:*

A prospective economic evaluation, conducted from a NHS and social care perspective, to provide estimates of cost utility and cost-effectiveness.

*Embedded Qualitative Study:*

Interview study with 20 participants at the end of the study to explore issues including acceptability, adherence, retention, attrition. Also, during the study, in the case of high loss-to-follow-up we will also undertake interviews with a sample of up to 10 participants in the intervention arm, who did not complete their 6 week follow up, to understand some of the reasons behind this.

**5. Lay Summary**

Social anxiety symptoms cause major problems for many people. The problems tend to last for years and people often do not seek help. Seeing a therapist for face-to-face cognitive behavioural therapy is recommended by NICE as the most effective approach for social anxiety disorder. We also know that many people have 'sub-clinical' social anxiety symptoms which cause them problems but are not severe enough to be diagnosed as 'social anxiety disorder'. We know many of these people turn to the internet to look for help where various unguided, self-directed internet-based tools are being made widely available - but there is very little evidence to say whether or not these are helpful.

We propose to test out the value of an internet self-help package (E-Couch) for sub-clinical social anxiety symptoms in the general population in a randomised controlled trial. We intend to provide E-Couch (or the ‘control’ website) to about 2000 people. These people will have social anxiety symptoms but not social anxiety disorder. For the people who are likely to have social anxiety disorder we will direct them to seek evidence-based help from the NHS, as recommended by NICE.

Over a period of 12 months we will measure the effect of using E-Couch self-help on social anxiety symptoms, as well as on quality of life, mental wellbeing, depression and general anxiety, and their use of other sources of help.

Harnessing the internet to help with a common problem among the general population has great potential to deliver low cost, widely accessible public mental health interventions. Our project will also give the opportunity to learn more about the value of using the internet to do this, and what the opportunities and challenges are - for researchers as well as for service providers and the public.

**6. The Public Health Approach**

The Internet is playing an ever more important role in our social and emotional lives. People go online in large numbers to find self-help solutions for a range of physical and mental health problems, and for broader health-related activities such as diet and exercise. Delivering self-help tools using online platforms offers a low cost and highly scalable approach that could potentially be harnessed by public health practitioners and policymakers seeking to tackle population-wide problems. However, the public health community has been slow to capitalize on this opportunity, and in general the pace of innovation has run ahead of both policymakers and evaluators. Thus, for many of these online self-help approaches, we do not have good evidence as to whether they are helpful or not (despite their wide use). Furthermore, there are also many unanswered generic questions about the value of the internet as a tool for delivering public health interventions.

In this study we will undertake a rigorous trial of an online self-help intervention for social anxiety symptoms in the general population. This is not about treating people with social anxiety disorder (for whom good treatments exist, and are available on the NHS, and recommended by NICE), but about testing the benefit of a novel public health approach to reduce the level of sub-clinical symptoms in the general population.

Building on our previous studies, a public health approach is appropriate for the following reasons: (1) sub-clinical social anxiety symptoms are very common and have a population distribution; (2) there are many people who could potentially benefit from interventions but who do not access them, and we will recruit directly from the population rather than through a service; (3) while there is evidence that therapist-guided internet-based social anxiety applications are effective, there is insufficient research to determine if fully self-directed interventions, which are becoming ever more available, are effective. Therefore research on online self-help is required because people are using it without strong evidence, and because of the potential cost-saving over a supported intervention, especially if applied at a population level.

Our intention is not only to learn about the effectiveness of our tool in reducing social anxiety symptoms, but also to learn more about the benefits and challenges of harnessing the internet to deliver public health interventions, and to explore methodological issues in doing large scale online public health trials. This will include exploring issues of recruitment, acceptability retention, and attrition.

**7. Our Team**

We are an interdisciplinary team with a track record of successful collaboration, and significant user involvement, based at two leading Universities (Oxford University and the Australian National University (ANU)). We have expertise in e-mental health and RCTs.

The research team is led by a public health physician with psychiatry training who is an expert in e-health (Powell), and the deputy lead is a academic consumer researcher, public policy fellow, registered psychologist and the Director of a global online mental health service (Griffiths). Powell is a public health physician and health services researcher at the University of Oxford who has been working in the area of e-health for 15 years and has led previous RCTs of internet-based interventions, including the PsyWell trial with Griffiths. Powell will take overall responsibility for the conduct of the trial. The project will join a portfolio of work on digital health hosted by the Oxford Collaboration for Leadership in Applied Health Research and Care (CLAHRC) of which Powell is an investigator. Griffiths is the deputy lead of the trial. She is the Director of ANU’s National Institute for Mental Health Research (NIMHR), a pioneer and world leading researcher in e-mental health, the Director of e-hub self-help services which delivers e-couch and a consumer researcher. Griffiths will participate in the trial design, provide advice on consumer involvement and will oversee the IT protocol development and trial activities at NIMHR.

The trial will be hosted by the Oxford University Primary Care Clinical Trials Unit and governed by the Standard Operating Procedures of this CTU which is nationally accredited. The CTU will provide our statistician (Ly-Mee Yu) is the lead statistician for the CTU; and our in-house health economist (Yaling Yang) works closely with the CTU. Our qualitative research will be supervised by Louise Locock, of the Oxford Health Experiences Research Group, also based in the Nuffield Department of Primary Care Health Sciences. Trial management and data management will be provided jointly by the Oxford Primary Care CTU and by the ANU team (Antony Bennett and Kylie Bennett), who have expertise and experience in delivering and project managing online trials and online interventions, and will cover all IT aspects

**8. Patient and Public Involvement**

We will build on our existing track records of significant public and patient involvement (both ANU and Oxford teams), to ensure user involvement throughout the trial process. In 2003 Griffiths founded the Depression and Anxiety Consumer Research Unit at ANU to promote the active participation of consumers in research, and she is an international pioneer in promoting the engagement of users as active participants in the research process. Powell has published on user involvement in e-health. We will have two lay representatives on our advisory group. These will be full members of our advisory group with written role descriptions and will be reimbursed for their time in accordance with INVOLVE guidelines. They will input into all aspects of the study - from advising on our final registered protocol and ethical approval submission documents, through to providing input into the final dissemination of the study findings to a range of audiences. Since receiving confirmation of funding we have had recruited one representative (Gareth Stephens - who undertook a similar role for NICE for their social anxiety guideline). We will work with MQ to ensure we have taken every opportunity to engage with MQ PPI activities. Our PPI work will be supported through a new post in the lead investigator’s department which has been created to facilitate PPI work across all our research activity (supported by CLAHRC funding). This person (Lynne Maddocks) took up her post in December 2014 and is familiar with this study.

**9. Context**

*Social anxiety*

Social anxiety is a major public health problem that often goes unrecognized and untreated. Epidemiological studies indicate that social anxiety disorder is one of the more common mental disorders with a one-year prevalence of 4%-6% [Griffiths, 2013], and a lifetime prevalence of up to 12% [Kessler, 2005]. It is characterized by an intense and persistent fear of being negatively evaluated in social or performance situations. Without treatment it generally follows an unremitting course and results in substantial disability and interferes with many areas of life. On average, compared to their counterparts, those with social anxiety disorder have lower levels of educational attainment, lower salaries, fewer friends, are less likely to marry, more likely to divorce, and less likely to have children [NICE guideline 2013]. Moreover, social anxiety disorder is associated with increased levels of depression, substance misuse, and suicidal ideation [see NICE guideline 2013; and Sareen et al., 2005]. The economic costs to society of the condition are substantial, with people with social anxiety disorder reporting more days off sick and impaired productivity due to symptoms, and also claiming more state benefits [NICE guideline 2013]. Many people with social anxiety disorder do not realize that they have a treatable condition. For this reason, many people do not seek help. Avoidance of social situations also reduces help-seeking. As a consequence there is a large unmet need in the general population of people who have a disabling condition for which treatment exists, but who do not get help [see Griffiths, 2013].

*The internet*

At the same time, we know that the internet is playing an increasingly important role in social life and in health in particular. Over 70% of the UK population uses the internet. The proliferation and widespread adoption of connected devices such as smartphones and tablet computers is leading to an ‘always on’ culture where people are becoming used to accessing online content for all sorts of public and private services, from any location. Increasingly health services are becoming digital – in the UK the NHS, through its website NHS Choices is transforming the healthcare environment and is increasingly providing digital services through a single portal. Our own work has shown that people using online mental health resources often have high levels of mental distress but are not receiving formal care elsewhere [Powell et al., 2003].

*E-therapy*

E-therapy has emerged as a new mode of treatment for a range of psychiatric and behavioural indications, and offers a low cost and highly scalable approach for common problems. Many of these e-therapy approaches use cognitive-behavioural models of treatment, and in becoming user-led and internet-based have their foundations in psycho-education, bibliotherapy and self-help. E-therapy can provide both supported and unsupported online information and interactive tools that users can use to work through a programme of treatment.

*Reviews of previous work*

A recent (2013) Cochrane review of Media-delivered CBT and BT interventions for anxiety [Mayo-Wilson & Montgomery, 2013] identified 15 studies of social anxiety compared with no treatment. The meta-analysis of these studies found a significant benefit of the interventions with a standard mean difference of 0.73 (95% confidence intervals 0.59-0.87), favouring the media-delivered therapy. A more recent meta-analysis reached a similar conclusion [Arnberg et al., 2014] finding a between group standardised mean difference of 0.85 (95% CI 0.66-1.05) for trials that compared the effectiveness of *therapist-guided* internet therapy for social anxiety, with a waiting list control group (n=8). The Cochrane review identified four studies that compared a media-delivered intervention with face-to-face therapy, finding no significant difference in treatment effect (standard mean difference 0.02, 95% confidence intervals -0.18-0.22). The authors note that the effect sizes (for all anxiety interventions, not only social anxiety disorder) for these media-based interventions were greatest for internet-delivered CBT. The authors noted that studies vary greatly in the level of therapist contact (if any) provided to the intervention group, and note that while therapist contact is beneficial in a trial setting it may not be practical for a pragmatic population-based approach to service delivery. The authors suggest that fully self-directed tools may have a role at one end of a stepped-care pathway.

*Current guidelines and service provision for social anxiety disorder*

We recognize that there are challenges associated with investigating the efficacy of an unproven, unguided, internet-based self-help for social anxiety symptoms. It is not our intention to deny people with social anxiety disorder the opportunity to receive a treatment with an established evidence base, to undertake a study that would conflict with national guidelines on the treatment of social anxiety disorder, to confuse participants in such a way that might delay them seeking other help, or to create problems if they receive other help in parallel. The National Institute for Health and Care Excellence (NICE) recently published their guideline on the assessment and treatment of social anxiety disorder [NICE, 2013]. This recommends that all NHS patients diagnosed with social anxiety disorder should be offered a course of individual cognitive behavioural therapy based on either the Clark and Wells or Heimberg model of treatment. The IAPT (Improving Access to Psychological Therapies) programme offers this therapy. If an individual declines individual CBT therapy then the NICE guideline recommends that they be offered supported self-help – e.g. advice from a therapist coupled with a workbook based on CBT principles. From 2015 the IAPT programme will, on a region by region basis, initiate a new therapist-supported internet-based treatment of SAD. Our final proposed methodology is very much informed by the above considerations and in particular by the NICE guideline; by the need for us to inform and direct people who are identified with a disorder to effective treatments; and by the treatments that will be available during the period of our proposed research. In order to avoid any confusion between what we are offering and what is available and recommended by NICE,we will clearly describe our intervention as a self-guided and unsupported self-help tool and not as therapy. For all these reasons this proposal is about offering self-help to people with sub-clinical social anxiety symptoms; people who meet criteria for social anxiety disorder (from their self-reported scale measurement) will be directed to seek other, more appropriate help according to NICE guidelines.

*Our approach*

Our proposal will address some of the limitations in the current evidence base which were identified in the Cochrane review. The review stated that large effectiveness studies are needed. The review stated that “few studies include follow-up after six months”. The review stated that previous work has often excluded large numbers of potential participants. The review also recommended that future work should examine mediators and moderators of adherence, should examine differences between responders and non-responders, and should pay systematic attention to any safety issues which have rarely if ever been examined in previous work. We will address all these issues in our proposal. Our methodology is also informed by our previous work. In a previous study using the CCBT intervention MoodGYM which was developed by the same ANU team as E-Couch, the applicants (led by Powell) showed that they could recruit 3070 participants to a randomized controlled trial in a period of two weeks using advertisements on the NHS Choices website (www.nhs.uk) [Powell et al., 2013]. Participants were invited to participate in a ‘mental fitness’ intervention and were allocated to MoodGYM or waiting list control condition. The study showed that relative to control, MoodGYM was effective at increasing mental wellbeing scores, and reducing depression and anxiety scores among a general population of internet users of a health website. A major lesson from this study was that internet trial recruitment tends to be “easy in, easy out” with high levels of attrition. The current proposal will therefore incorporate a number of low cost measures that have previously been shown to combat attrition in internet studies including the use of automated text (SMS) messages.

**10. Study Design**

*Summary*

A large, pragmatic, double-blind, randomised controlled trial of the internet-based self-directed E-Couch social anxiety module versus a waiting list control condition, for the treatment of sub-clinical social anxiety symptoms, among a general population sample recruited using direct-to-consumer advertisements placed on national websites.

*Our intervention*

E-Couch (https://ecouch.anu.edu.au) is an online toolkit of self-directed modules covering common mental health problems including social anxiety, generalized anxiety, depression, relationship breakdown, and loss & grief. It was developed by researchers at the Australian National University (ANU) who are co-applicants on this proposal. The social anxiety module is based on cognitive behavioural therapy principles and includes components of known effectiveness in face-to-face therapy. This module contains a literacy section and 5 toolkits comprising exposure practice, cognitive restructuring (modifying your thinking), attention practice, social skills training and relaxation. E-Couch is designed to be completed at the participant’s own pace. It is free to use, browser-based and widely accessible on a range of connected devices. Analysis of unpublished data collected from spontaneous visitors from around the world to the publicly accessible E-Couch shows a statistically significant reduction in social anxiety symptoms among visitors to the E-Couch social anxiety toolkit. In a small (n=63) laboratory-based randomized controlled trial, Bowler et al. (2012) showed that the social phobia modules of the E-Couch toolkit which are described above resulted in a significantly greater reduction in social anxiety than control (Effect size d=1.0). In addition, the E-Couch toolkit was as efficacious as a cognitive bias modification intervention at reducing levels of social anxiety, trait anxiety, and depression, relative to a no intervention control group [Bowler et al., 2012]. Although there was no formal face-to-face therapeutic input in this context, the study did ensure participants completed the set modules. Thus, to date, there have been no controlled studies of the intervention with a fully self-guided approach. Moreover, to our knowledge there has only been one trial comparing therapist-guided with unguided (or self-guided) internet-based treatment of social anxiety. Undertaken by Titov et al (2008), an intent-to-treat analysis found that the unguided intervention was not effective relative to control. However, there was evidence that the program was helpful for the participants who completed the intervention. In summary, there is evidence that therapist-guided internet-based cognitive behavioural therapy for social anxiety disorder is effective; however, there is a lack of evidence on the value of self-guided internet interventions such as E-Couch with no therapist contact. This latter approach requires further research particularly given the potential resource savings over the therapist-guided approach if widely disseminated across the population, and the fact that other fully self-guided approaches are being promoted without evidence (for example, via mobile apps).

The E-Couch toolkit is available online for anyone who registers to use it (<https://ecouch.anu.edu.au>). For this study a social anxiety specific tool will be packaged using the e-couch social anxiety content to create the intervention. We do not anticipate high levels of contamination in the control arm – we will not call the intervention ‘e-couch’ in this study (it will be given a new, study-specific name), and e-couch (and the social anxiety elements within the toolkit) are not widely known or used by the UK population. We will measure contamination and use of any other online social anxiety tools through participant self-report at the time of final follow-up, using a bespoke questionnaire asking respondents which of a list of online interventions they have or have not also accessed during the study period. This is a pragmatic study and we would expect some participants to also be seeking other self-help, in both arms of the study.

*Control group*

The control group receive no intervention and are placed on a waiting list to receive the intervention at the end of the trial.

*Study population*

Internet users who consent to take part in a study for self-help with sub-clinical social anxiety symptoms. To be eligible they must have some level of social anxiety symptoms, defined as scoring 13 or more on the SPIN instrument (the population mean is generally 11 or 12, so the score of 13+ was chosen to pick up people with mild symptoms. People who score over 20 are more likely to have social anxiety disorder).

This is an amendment to the original protocol that required eligible participants to score 13-19 on the SPIN instrument. However it became clear after 6 months of recruitment that very few individuals score in this range, and most people with any level of social anxiety symptoms score more highly.

*Recruitment*

We will use advertisements placed on websites including NHS Choices, the NHS website, which receives more than 25 million unique visits per month making it one of the most visited websites in the UK and certainly the most popular health website. It is also a widely trusted source of health information and the NHS branding is known to facilitate recruitment to research studies [Todkill & Powell, 2013]. As noted above, the current team used advertisements on the NHS Choices website very successfully in a previous trial of an e-therapy intervention, recruiting 3070 individuals in a 2-week period [Powell et al., 2013].

*Eligibility screening and consent*

Individuals expressing an interest in the online advert by clicking on a web link will be directed to a University website containing full information on the trial and the opportunity to ask questions of the study team by email or telephone.

They will be invited to complete screening questions which will determine whether they are eligible for participation: they will need to confirm the following: a working email address (to respond to a confirmation email and receive reminders), a mobile phone number (to receive text reminders), and that they are aged 16 or over and resident in the UK. We will exclude people who are currently receiving therapist-guided treatment for social anxiety disorder. If they meet these criteria, they will be asked to provide online consent. In line with our public health approach and the pragmatic nature of this study, our inclusion criteria have been kept very broad. Having given informed consent, participants will know that they are free to withdraw at any time without giving a reason and without it affecting their care.

In providing consent, participants will give their agreement to complete two screening measures (the SOPHS and the SPIN) and to be randomized to one of two conditions (E-Couch intervention or waiting list control). The consent will also give their agreement to be contacted by email and text message to send them study reminders. Separate consent will be sought for any qualitative interview as part of the study. Participants will be eligible if they score 13 or more on the SPIN.

We do not want to delay any form of help-seeking and all participants with a score>19 on the SPIN will be given advice to seek therapist-guided help from the NHS (including IAPT) as their scores indicate they may have social anxiety disorder (note that we have excluded people currently receiving treatment).

In order to inform future research and to support some exploratory analyses of our data we will include the new SOPHS social phobia screener (<http://nimhr.anu.edu.au/mental-health-measures/social-phobia-screener-sophs>) at this screening stage, although it will not be used for screening at the time (only in post hoc analyses).

*Initial trial flow and collection of baseline measures*

In addition to the SPIN and SOPHS, eligible participants will be required to complete a battery of self-report baseline questionnaires, and in line with all other trial procedures these will be collected online through the trial portal. These questionnaires will collect demographic characteristics (age, sex, education, employment, marital status etc.), and we will measure social anxiety symptoms (SPIN and SOPHS as above, and Brief Fear of Negative Evaluation BFNE-S scale), depression (CES-D), mental wellbeing (SWEMWBS), quality of life (SF-36), and current use of health services and psychological interventions (including self-help).

Once baseline measures are completed, eligible participants will be randomized in a 1:1 ratio with no stratification, using computer generated random number sequence run through an automatic online programme. Participants in the intervention arm will be given access to a password-protected website and will be encouraged to access and use the self-guided intervention over a period of up to 6 weeks (although they can work through the intervention at their own pace). They will receive one text message within 24 hours of randomisation to thank them for participating and to remind them to access the intervention, and one further text message towards the end of the 6 week period to thank them again and to remind them that they will shortly be asked to complete follow-up measures. They will also receive three email reminders during the six-week period (both intervention and control will receive the reminders – at 1 week, 3 weeks, and 5 weeks). Participants in the Control arm will receive the same reminders and will be given access to the intervention after final follow-up (12 months). Participants in the intervention arm will be offered continued access to the intervention once the trial is completed.

*Follow-up measures*

Self-report follow-up measures of social anxiety symptoms (SPIN, SOPHS, and Brief Fear of Negative Evaluation BFNE-S scales), depression (CES-D), mental wellbeing (SWEMWBS), quality of life (SF-36), and current use of health services and psychological interventions, and time off from work or study due to psychological problems will be taken at 6 weeks (following completion of the self-guided intervention), 3 months, 6 months, and 12 months. At each follow-up point they will receive notification of follow-up measures by email and by text message, and if measures are not completed they will receive up to three follow-up emails.

We will also record usage data (total number of logins, total page views, time on site). We will record measures of adherence to the intervention (number of components of E-Couch completed) and attrition from the trial (loss-to-follow-up at each time point). We will also record any safety (adverse) events (captured through monitoring participant feedback, and through questions at each follow-up period). We do not anticipate adverse events in a trial of a low intensity online self-help tool for a subclinical population. However our adverse event monitoring will include assessment of feedback from participants (including routine questions asked at each follow-up period as well as ad hoc correspondence such as emails or telephone calls) to see if these report an issue such as distress or anxiety caused by the intervention. In the first instance any possible adverse event will be reviewed by the study team (trial manager in discussion with lead investigator), and immediate action taken (such as withdrawing a participant who reports distress). If the event is classified as an adverse event it will be referred to the Trial Steering Committee (with independent chair) who will also have the role of Data Monitoring Committee for their consideration.

*Primary outcome measure*

This will be the change in self-reported social anxiety score using the 17-item SPIN. This measure has been shown to have adequate internal consistency, test-retest reliability, construct validity and sensitivity to change following intervention [Letamendi et al., 2009].

*Secondary outcome measures*

These will include social anxiety measured using the FNE scale; depression (CES-D); mental wellbeing (SWEMWBS); quality of life (SF-36); and safety events.

*Statistical methods*

The primary statistical analysis will be carried out on the basis of intention-to-treat (ITT). We will endeavour to obtain full follow-up data on every participant to allow full ITT analysis, but we will inevitably experience the problem of missing data due to withdrawal, loss to follow up, or nonresponse to some questionnaire items. The results from the trial will be prepared as comparative summary statistics with 95% confidence intervals. All the tests will be done at a 5% two-sided significance level. The study results will be reported in accordance with the CONSORT (Consolidated Standards of Reporting Trials) 2010 statements. A full statistical analysis plan will be prepared before the recruitment starts.

To understand participant disposition, a flowchart showing numbers of participants screened, randomised and those who are available for the final analysis populations will be constructed. Reasons for withdrawal, exclusions or losses to follow-up will be shown at each stage during the study by intervention group.

A mixed effects model will be used to analyse the primary outcome (SPIN score), utilising data collected at 6 weeks, 3, 6 and 12 months from randomisation. An interaction between time and randomised group will be fitted to allow estimation of treatment effect at each time point. The model will adjust for the SPIN score at baseline gender.

Analysis of secondary outcomes, such as scores to measure social anxiety (FNE scale), depression (CES-D), mental wellbeing (SWEMWBS), and quality of life (SF36) will be similar to the method used for the primary outcome. Safety data will be analysed using Fisher’s exact or Chi-squared tests. Other binary outcomes will be analysed using generalised linear model with log link function.

For both primary and secondary outcomes we will include subgroup analyses of baseline factors which may influence the intervention effect (moderators). Baseline subgroups will be compared by including interaction terms in the model (baseline subgroup by treatment group interaction). Baseline factors to be compared include: age; gender; educational level; baseline level of sub-clinical social anxiety symptoms

Mediation analyses will be undertaken to determine if there is a dose-response effect of the intervention with more interaction with the website equating to higher levels of treatment effect (examining ‘dose’ in terms of number of total usage of site and numbers of modules completed). For both groups, their usage of the intervention will be recorded (total number of logins to site, total time on site, total number of modules completed). These will be tallied so that the total at 6 weeks, 3, 6 and 12 months can be computed and used in the analysis. We will also undertake mediation analyses to explore the effect of receiving other help for sub-clinical social anxiety symptoms during the trial on the treatment effect observed. If the program is effective those randomised to the control condition may be more likely to seek additional help elsewhere – a potential source of confounding which has the potential to reduce the observed effect. In addition, we will use a mediation model (Emsley et al., 2010; Mackinnon et al., 2002) to assess the influence of adherence/compliance to treatment outcome.

Other prespecified analyses will explore factors influencing response and non-response, and influencing adherence to the intervention (in terms of numbers of modules completed) and attrition from the trial (i.e. loss to follow-up). Factors which we will examine will include gender, age, ethnicity, other sources of help/support, severity of symptoms, etc. Exploratory analyses of usage data will allow us to examine any dose response effect of the intervention, and to examine whether certain components of the intervention have more benefit than others.

We will explore the mechanism of missing data by looking for associations between participant characteristics and the likelihood of non-response to questionnaires at different time points. This can be done using a regression model for binary outcomes (1=response; 0=non-response) with independent variables measured at baseline (gender, age, educational attainment, marital status, baseline questionnaire scores (FNE, SPIN, CES-D, SWEMWBS, SF-36)). If significant associations are observed this lends weight to a missing at random (MAR) assumption. The primary outcome analysis will utilise a mixed effects model which implicitly accounts for data under a MAR assumption.

We will do sensitivity analysis for the primary outcome using methods which do not assume a MAR mechanism such as pattern-mixture models, to assess the robustness of this assumption. If different results are obtained from a pattern-mixture model compared to the mixed effects model then it is likely that the MAR assumption is not valid. As part of our exploration of treatment effects we will also produce a per-protocol or completer analysis with appropriate caveats.

*Sample size*

Although, previous studies and the recent systematic review have suggested a large treatment effect observed with supported internet-delivered interventions, we believe this treatment effect is too optimistic for a self-guided treatment, especially in a sub-clinical population group, and is also likely to be smaller in pragmatic settings. For this reason, we intend to recruit 2104 participants (i.e. 1052 per group) to this trial, which will provide 90% power to detect a small between group effect size of Cohen's d=0.2 at 5% two-sided significant level. A small effect like this may not be that important to the individual but is potentially important in population terms, in a public health intervention. This sample size has also accounted for a high level of potential attrition of up to 50% (obviously we will aim to mitigate this through frequent email and text message reminders but we need to be realistic about self-guided internet interventions and the likely level of attrition). We believe this is entirely feasible and achievable using our previous recruitment strategy and given the high population prevalence of social anxiety symptoms.

*Economic evaluation*

A prospective economic evaluation, conducted from a NHS and social care perspective, will be integrated into the trial to assess cost and cost-effectiveness of the self-guided internet-based intervention for people with pre-clinical social anxiety symptoms. Primary research methods will be followed to estimate the costs of delivering the intervention, including development and maintenance costs for the website, advertisement costs, and the costs of monitoring participants for safety or other reasons. Taking a NHS and social care perspective means that broader health service and social care utilization costs will be included in the primary economic evaluation. Health service and social care utilisation data will be collected through participant self-report questionnaires administered at baseline, and each follow-up time point. Unit costs for service utilisation will be derived from standard national sources such as Unit Costs of Health and Social Care (PSSRU), NHS reference cost etc. ,and estimated in line with best practice. Costs will be standardised to current prices where possible. Given the fact that the intervention will target people who are unlikely to pursue help from NHS or social care due to the sub-clinical mild nature of the symptoms, we don’t expect the intervention to have significant impact on health and social care costs. On the other hand, these symptoms may impact on people’s social life which leads to loss of productivities due to leaves from work, study, or absent from leisure activities. We will collect data to assess this wider social impact.

The effectiveness of the intervention will be measured using Quality-Adjusted Life Years (QALYs) using the under the curve approach. Quality of life will be measured at baseline, 6 weeks, 3 months, 6 months, and 12 months using the SF-36. The data will be converted into SF-6D health utilities using established UK based utility algorithms and combined with time duration data (e.g. 6 weeks, 3 months, and 6 months according to the follow up time points from baseline) calculate quality-adjusted life years (QALYs). We are using the SF-6D (derived from SF36), which is a widely accepted generic preference-based measure because we have problems to integrate an online version of the EQ5D and portal to our trial design

The results of the economic evaluation will primarily be expressed in terms of incremental cost per QALY gained between the intervention and control groups. Missing data for both costs and SF-36 will be explored using the same strategies of the statistical analysis. Multiple imputation approach will be used to handle missing data. Non-parametric bootstrap estimation will be used to calculate 95% confidence intervals for mean difference of cost and QALYs between the trial groups, and incremental cost effectiveness ratios. Regression will be used to obtain incremental cost-effectiveness ratios after controlling for baseline respondent’s characteristics. A series of sensitivity analyses will explore the implications of uncertainty on the incremental cost-effectiveness ratios and will consider the broader issue of the generalisability of the results. Cost-effectiveness acceptability curves will be constructed using the net benefits approach.

*Qualitative study*

We will collect qualitative survey data (free text box) on initial views and experiences of the intervention for all participants in the intervention arm at six weeks. These data will be analysed using content analysis and where appropriate thematic analysis. In addition, a qualitative interview study will be conducted with a subset of approximately 20 participants in the intervention arm at 12 months and with those who withdraw from the trial. The latter interviews will focus on reasons for withdrawal. For those who complete the trial, a maximum-variation sampling technique will be used to select a diverse sample of potential interviewees based on demographic information and degree of completion of the intervention. Interviews will be audiorecorded, they will last approximately one hour and will be conducted in the participant’s home or (if inconvenient or impractical to conduct a home visit) by telephone. They will follow a semi-structured topic guide that will be piloted. Interview transcripts will be analysed iteratively and thematically using a constant comparative approach, to explore issues related to acceptability, usability, adherence, attrition, and perceived value and impact of the intervention. This investigation will be led by the internationally leading Health Experiences Research Group in the lead applicant's department.

In the case of high loss-to-follow-up we will conduct brief additional telephone interviews with up to 10 participants from the intervention arm, who did not complete their 6 week follow up, to help identify the reasons for loss to follow up.

All participants taking part in the telephone interviews will receive £15 shopping gift voucher.

**11. Ethics and Governance**

We will obtain all ethics and governance permissions prior to project start and have significant experience in doing so previously. The trial protocol will be registered with ISRCTN. One of the key ethical issues for us will be to ensure we comply with the NICE guideline on social anxiety disorder, and as stated above if individuals likely to have social anxiety disorder (SPIN=19+) are identified then we will make sure they have clear advice to seek help from existing services for the NICE-recommended treatments. .We will collect data on their use of health services throughout the trial period.

We will require University ethics committee approvals from the University of Oxford and the Australian National University. We have received confirmation that we do not require NHS ethics approval (confirmed with the Health Research Agency (HRA)).

**12. Quality Assurance and Reproducibility**

The University of Oxford will take on the role of study sponsor for this study. Given the nature of this study no formal site monitoring will be conducted. Central monitoring of the progress of the study and the data quality will be provided by the Oxford Primary Care Clinical Trials Unit (PC-CTU) Quality Assurance Manager and Data Manager. An independent joint Trial Steering Committee (which in common with many ‘low risk’ trials will also have the role of Data Monitoring Committee) with independent chair and lay representation will be established to provide oversight of the conduct of the study and of the data emerging. The Committee will meet on a regular basis 6-monthly and provide advice to the Trial Management Team, which will meet monthly (with Skype to include ANU members). A representative of the Sponsor and the Funder will be invited to all Trial Steering Committee Meetings and informed of any reports to the Trial Management Team.

For reproducibility: The trial will be registered on ISRCTN prior to commencement and we will publish our final protocol (e.g. in BioMedCentral). Our Statistical Analysis Plan (SAP) will be registered and available on request. Our findings will be written up in accordance with the CONSORT statement, and in line with the Nature Publishing Group reporting checklist for Life Sciences articles, and in line with ICJME guidelines on authorship.

**13. Data Management**

Data will be collected from participants once informed consent has been received. All data will be kept confidential and held securely. The collection, transfer and storage of personal data will comply with the PC-CTU Standard Operating Procedures (SOPs), which are fully compliant with the Data Protection Act 1998 and Good Clinical Practice (GCP). Patient identifiable information (the email address and mobile telephone number used for sending reminders) will be held separate to other data during the course of the study. Otherwise, participant data will be identified only by Study ID number.

Data obtained through participant self-completion on the trial portal at baseline and all follow-up points will initially be collected and stored on ANU servers physically located in a controlled access room, with network and machine level firewalls. Once the trial is complete (last participant completed final follow-up) the trial dataset will be transferred to the Oxford clinical data management system (OpenClinica Enterprise) for analysis and subsequent archiving, using a secure encrypted (SSL) file download (access restricted by IP address). Once transferred, the database will be hosted on servers fully managed by the University of Oxford. The final anonymous trial dataset will be securely electronically archived in accordance with PC-CTU SOPs, initially for a minimum of five years. Throughout the study data monitoring and validation checks will be carried out by the Primary Care Clinical Trials Unit (PC-CTU) Quality Assurance Manager.

We will be pleased to make our final anonymous dataset available for subsequent use by others, in line with our data sharing policy, subject to ethical approval, and within the limits of participant consent. Any request for either complete or partial datasets will be considered in line with PC-CTU SOPs. The PI will consider all requests and data will be provided on the understanding that it is used for the purpose outlined in the request, that use of the dataset does not create any claim in ownership of the dataset. The PI retains ownership of the dataset and that it will not be transferred to a third party without agreement from the PI. Intellectual Property in the dataset will be jointly owned by the University of Oxford and The Australian National University, and will be detailed in a Research Collaboration Agreement between the institutions.

**14. Dissemination**

At the end of the study we will disseminate our findings widely. It is well established that getting evidence into practice is not achieved solely by producing academic outputs. To achieve effective knowledge translation we need to produce multiple outputs, in multiple formats, for multiple audiences, and we need active, ongoing engagement with stakeholders. Therefore, in addition to peer-reviewed publications (open access where possible) and conference presentations, and a full report to the funder (MQ), we will also produce three summaries of every output: for lay, policy and practitioner audiences. These will be disseminated widely as targeted hard copies to key stakeholders, include patient organisations, and available for free download from our website. Findings will also be shared on social media and through press releases (in conjunction with MQ). We will also present findings at a final workshop targeting key audiences including patients, public and their representatives.

At the end of the trial ANU are well placed to ensure the continued implementation and delivery of the self-directed version of the E-Couch social anxiety application, free of charge to the general public, should it prove effective. ANU have provided a high profile global online mental health service for 13 years.

**15. References**

Arnberg, F. K., Linton, S. J., Hultcrantz, M., Heintz, E., & Jonsson, U. (2014). Internet-delivered psychological treatments for mood and anxiety disorders: A systematic review of their efficacy, safety, and cost-effectiveness. PloS One, 9(5), e98118.

Bowler J.O., Mackintosh B., Dunn B.D., Mathews A., Dalgleish T., Hoppitt L. (2012). A comparison of cognitive bias modification for interpretation and computerized cognitive behavior therapy: effects on anxiety, depression, attentional control, and interpretive bias. Journal of Consulting and Clinical Psychology. 80(6):1021–1033.

Emsley et al (2010). Modelling mediation and moderation of treatment effects in randomised controlled trials of complex interventions. Statistical Methods in Medical Research, 19, 237-270

Griffiths, K.M., Crisp, D., Christensen, H., Mackinnon, A.J., Bennett, K. (2010). The ANU WellBeing study: A protocol for a quasi-factorial randomised controlled trial of the effectiveness of an Internet support group and an automated Internet intervention for depression. BMC Psychiatry 10:20.

Griffiths KM, Mackinnon AJ, Crisp DA, Christensen H, Bennett K, Farrer L. (2012). The effectiveness of an online support group for members of the community with depression: a randomised controlled trial. PLoS ONE. 7(12):e53244.

Griffiths KM. Towards a framework for increasing help-seeking for social anxiety disorder. (2013). Aust N Z J Psychiatry. 47(10):899-903.

Kessler, R. C., Berglund, P., Demler, O., Jin, R., Merikangas, K. R., & Walters, E. E. (2005). Lifetime prevalence and age-of-onset distributions of DSM–IV disorders in the National Comorbidity Survey Replication. Archives of General Psychiatry. 62:593–602.

Letamendi AM, Chavira DA and Stein MB. (2009). Issues in the assessment of social phobia: a review. Israel Journal of Psychiatry and Related Sciences. 46:13-24.

MacKinnon DP, Lockwood CM, Hoffman JM, West SG, Sheets V. (2002) A comparison of methods to test mediation and other intervening variable effects. Psychological Methods, Vol 7(1), 83-104.

Mayo-Wilson E, Montgomery P. Media-delivered cognitive behavioural therapy and behavioural therapy (self-help) for anxiety disorders in adults. (2013). Cochrane Database Syst Rev. 14:CD005330.

NICE, National Institute for Health and Care Excellence. (2013). Social anxiety disorder: recognition, assessment and treatment. (Clinical Guideline 159) http://www.nice.org.uk/guidance/cg159

Powell J, McCarthy N, Eysenbach G. (2003). Cross-sectional survey of users of Internet depression communities. BMC Psychiatry. 3:19.

Powell J, Hamborg T, Burls A, Stallard N, McSorley J, Bennett K, Griffiths K, Christensen H. (2013). The PsyWell study: Randomised Controlled Trial of an intervention to improve mental wellbeing in the general population. Journal of Medical Internet Research. 15(1):e2.

Rapee RM, Abbott MJ, Baillie AJ and Gaston JE. (2007). Treatment of social phobia through pure self-help and therapist-augmented self-help. The British Journal of Psychiatry. 191: 246-52.

Sareen J, Cox BJ, Afifi TO, et al. (2005). Anxiety Disorders and Risk for Suicidal Ideation and Suicide Attempts: A Population-Based Longitudinal Study of Adults. Arch Gen Psychiatry. 62(11):1249-1257.

Todkill T, Powell J. Participant experiences of an internet-based intervention and randomised control trial: interview study. (2013). BMC Public Health 13:1017
