# Supplementary material for: Effectiveness and Cost-Effectiveness of a Self-Guided Internet Intervention for Social Anxiety Symptoms in a General Population Sample: Randomized Controlled Trial
Source: J Med Internet Res. 2020 Jan 10;22(1):e16804. doi: 10.2196/16804 (PMC6996778; doi:10.2196/16804)
Supplement: Multimedia Appendix 2 [file jmir_v22i1e16804_app2.docx]

**Multimedia appendix 2: sensitivity, subgroup and additional analyses (including e-tables and e-figures referred to in main text)**

**Effectiveness of a self-guided internet intervention for social anxiety symptoms: population based randomized trial.**

Contents

[1.1 Number analysed 3](#_Toc5268261)

[1.1.1 Comparison of baseline characteristics for participants lost to follow-up at 6 weeks 3](#_Toc5268262)

[1.2 Sensitivity analyses 5](#_Toc5268263)

[1.2.1 Completer-only analysis including participants returning all spin-17 outcome measures at all time points 5](#_Toc5268264)

[1.2.2 Completer-only analysis including participants returning all spin-17 outcome measures at 6 weeks and 12 months only 6](#_Toc5268265)

[1.2.3 Primary analysis of SPIN-17 adjusting for covariates found to be associated with missing SPIN-17 at 6 weeks 7](#_Toc5268266)

[1.2.4 Missingness pattern of the primary outcome 8](#_Toc5268267)

[1.2.5 Primary analysis assuming plausible arm specific differences (assuming data MNAR for SPIN-17 at 6 weeks) 10](#_Toc5268268)

[1.2.6 System glitch at 6 months follow-up 11](#_Toc5268269)

[1.3 Subgroup analyses 12](#_Toc5268270)

[1.3.1 Subgroup analysis with change in SPIN-17 score at 6 weeks as outcome 12](#_Toc5268271)

[1.3.2 Subgroup analysis with change in fear of negative evaluation (BFNE-S) at 6 weeks as outcome 13](#_Toc5268272)

[1.3.3 Subgroup analysis with change in depression (CES-D) at 6 weeks as outcome 14](#_Toc5268273)

[1.3.4 Subgroup analysis with change in mental well-being (SWEMWBS) at 6 weeks as outcome 15](#_Toc5268274)

[1.4 Additional analysis 16](#_Toc5268275)

[1.4.1 Exploring difference in trend in SPIN-17 between randomized groups 16](#_Toc5268276)

[1.4.2 Exploring adherence to E-couch social anxiety module 16](#_Toc5268277)

[1.4.3 Description of Usage data, Adherence and Other help received (self-help and help from a health professional) 18](#_Toc5268278)

[1.4.4 Dose-response effect of intervention 19](#_Toc5268279)

[1.4.5 Exploring if some modules are more beneficial than other in terms of change in SPIN-17 score 21](#_Toc5268280)

[1.4.6 Mediation analysis 22](#_Toc5268281)

Contents of Tables

[e-Table 1 Number of participants lost to follow-up, withdrew, or missing primary outcome by randomized group 3](#_Toc27578890)

[e-Table 2 Comparisons of the lost to follow-up at each time point for E-Couch vs Control 3](#_Toc27578891)

[e-Table 3 Baseline characteristics stratified by non-missing/non-missing SPIN-17 at 6 weeks 4](#_Toc27578892)

[e-Table 4 Descriptive summaries of mean and change in SPIN-17 score at each time point for each group for the completer population by randomized group 5](#_Toc27578893)

[e-Table 5 Adjusted estimates from mixed effect model of change in SPIN-17 score at 6 weeks and 12 months for completers 6](#_Toc27578894)

[e-Table 6 Descriptive summaries of mean and change in SPIN-17 score at each time point for each group for the completer population (6 weeks and 12 months) by randomized group 6](#_Toc27578895)

[e-Table 7 Adjusted estimates from mixed effect model of change in SPIN-17 score at 6 weeks and 12 months for completers (at 6 weeks and 12 months only) 7](#_Toc27578896)

[e-Table 8 Adjusted estimates from mixed effect model of change in SPIN-17 at 6 weeks and 12 months Adjusted for covariates predicting missingness 7](#_Toc27578897)

[e-Table 9 Missingness pattern for the SPIN-17 scores at the follow-up time points by randomized group 8](#_Toc27578898)

[e-Table 10 Sensitivity analysis using MNAR assumption for the effect of E-Couch on SPIN-17 at 6 weeks 10](#_Toc27578899)

[e-Table 11 Proportion of participants returning 6 month follow-up questionnaires within 4 weeks by randomized group 11](#_Toc27578900)

[e-Table 12 Adjusted estimates from mixed effect model of change in SPIN-17 at 6 weeks with time fitted as a linear covariate in the model 16](#_Toc27578901)

[e-Table 13 Summary of total modules completed over the study period 16](#_Toc27578902)

[e-Table 14 Adjusted Poisson regression model for adherence in participants who received E-Couch intervention 17](#_Toc27578903)

[e-Table 15 Summary of usage data, adherence and other help received by randomized group 18](#_Toc27578904)

[e-Table 16 Dose-response effect of E-Couch on change in SPIN-17 score at 6 weeks 19](#_Toc27578905)

[e-Table 17 Dose-response effect of E-Couch on change in SPIN-17 score at 12 months 20](#_Toc27578906)

[e-Table 18 Summary statistics of SPIN-17 score for each module by time point by status of module 21](#_Toc27578907)

[e-Table 19 Estimates of indirect effect for mediators at 6 weeks for change in SPIN-17 outcome at 12 months from mediation analysis 22](#_Toc27578908)

Contents of Figures

[e-Figure 1 Pattern mixture model results for SPIN-17 at 6 weeks 9](#_Toc27578917)

[e-Figure 2 Subgroup Forest plot for change in SPIN-17 at 6 weeks for E-Couch versus Control 12](#_Toc27578918)

[e-Figure 3 Subgroup Forest plot for change in BFNE-S at 6 weeks for E-Couch versus Control 13](#_Toc27578919)

[e-Figure 4 Subgroup Forest plot for CES-D for E-Couch versus Control 14](#_Toc27578920)

[e-Figure 5 Subgroup Forest plot for SWEMWBS for E-Couch versus Control 15](#_Toc27578921)

## Number analysed

e-Table 1 provides of the number of participants that were lost to follow-up by randomized group at each time point (all tables in this multimedia appendix have the prefix “e-“). Comparison of lost to follow-up between randomized groups is shown in e-Table 2, indicating the Odds of lost to follow-up was higher in the E-Couch group compared to the Control group. This was consistent at all time points.

e-Table 1 Number of participants lost to follow-up, withdrew, or missing primary outcome by randomized group

|  | E-Couch Social Anxiety Module | Control | Total Randomized |
| --- | --- | --- | --- |
|  | N=1061 | N=1061 | N=2122 |
| Excluded due to ineligibility^a^ | 3 | 3 | 6 |
| Number randomized and eligible | 1058 | 1058 | 2116 |
| Number of participants lost to follow-up, withdrew, or missing primary outcome over the 12 months of the study |  |  |  |
| Baseline, n(%) | 0 (0) | 0 (0) | 0 (0) |
| 6 weeks follow-up, n(%) | 643 (60.8) | 268 (25.3) | 911 (42.9) |
| 3 months follow-up^b^, n(%) | 884 (83.6) | 630 (59.6) | 1514 (71.6) |
| 6 months follow-up^b^, n(%) | 793 (75.0) | 436 (41.2) | 1229 (58.1) |
| 12 months follow-up, n(%) | 709 (67.0) | 348 (32.9) | 1057 (50.0) |

^a^Age recorded was implausible and below 18 years of age

^b^ There was an administrative error with the software sending automatic emails regarding questionnaire surveys which stopped working for about a month before it was spotted. Once this glitch was fixed the response period to have a ‘catch-up’ was extended from 4 weeks to 8 weeks for those participants who were missed but most were too late. As a consequence, follow-up data of outcome measures at 3 and 6 months data were very low.

e-Table 2 Comparisons of the lost to follow-up at each time point for E-Couch vs Control

|  | E-Couch Social Anxiety Module vs Control |
| --- | --- |
|  | **Odds Ratio of lost to follow-up [95% CI], P-value** |
| 6 weeks follow-up | 4.57 [3.79 to 5.50], P<0.0001 |
| 3 months follow-up | 3.45 [2.82 to 4.23], P<0.0001 |
| 6 months follow-up | 4.27 [3.55 to 5.14], P<0.0001 |
| 12 months follow-up | 4.14 [3.46 to 4.97], P<0.0001 |

### Comparison of baseline characteristics for participants lost to follow-up at 6 weeks

e-Table 3 shows summary statistics of baseline characteristics stratified by not missing/missing SPIN-17 score at 6 weeks. Assessment of which variables were associated with missing SPIN-17 score at 6 weeks was carried out using logistic regression. The variables that were associated with missing SPIN-17 score at 6 weeks were; younger age (P<0.0001), marital status (not married) (P=0.0077), higher BFNE-S score (P=0.0370), higher CES-D (P=0.0398), higher SF-36 PCS (0.0050) and lower SF-36 MCS (P=0.0023).

e-Table 3 Baseline characteristics stratified by non-missing/non-missing SPIN-17 at 6 weeks

| Baseline Characteristics | Not Missing  (N = 1205) | Missing  (N = 911) | P-value^a^ |
| --- | --- | --- | --- |
| Age (years) |  |  | <0.0001 |
| Mean (SD) | 38.5 (14.1) | 35.4 (13.1) |  |
| [Range] | [18 to 84] | [18 to 81] |  |
| Gender |  |  | 0.3708 |
| Female, n(%) | 977 (81.1) | 721 (79.1) |  |
| Male, n(%) | 220 (18.3) | 180 (19.8) |  |
| Other, n(%) | 8(0.6) | 10 (1.1) |  |
| Marital Status |  |  | 0.0077 |
| Married or in a civil partnership, n(%) | 391 (32.5) | 247 (27.1) |  |
| Not married, n(%) | 810 (67.2) | 662 (72.7) |  |
| Missing, n(%) | 4 (0.3) | 2 (0.2) |  |
| Education |  |  | 0.2568 |
| Degree, n(%) | 612 (50.8) | 440 (48.3) |  |
| No Degree, n(%) | 593 (49.2) | 471 (51.7) |  |
| Employment Status |  |  | 0.0657 |
| Employed, n(%) | 682 (56.6) | 551 (60.5) |  |
| Unemployed, n(%) | 514 (42.7) | 352 (38.6) |  |
| Missing, n(%) | 9 (0.7) | 8 (0.9) |  |
| Income |  |  | 0.0851 |
| ≤ £25,000, n(%) | 858 (71.2) | 617 (67.7) |  |
| > £25,000, n(%) | 347 (28.8) | 294 (32.3) |  |
| Ethnicity |  |  | 0.7937 |
| White, n(%) | 1047 (86.9) | 788 (86.5) |  |
| Non-white, n(%) | 158 (13.1) | 123 (13.5) |  |
| Social Phobia Inventory (SPIN-17) |  |  | 0.2398 |
| Mean (SD) | 39.4 (13.5) | 40.1 (13.0) |  |
| [Range] | [13 to 68] | [13 to 68] |  |
| Mental well-being score (SWEMWBS) |  |  | 0.0822 |
| Mean (SD) | 17.8 (3.3) | 17.6 (3.1) |  |
| [Range] | [7.0 to 35.0] | [7.0 to 30.7] |  |
| Brief fear of negative evaluation score (BFNE-S) |  |  | 0.0370 |
| Mean (SD) | 22.2 (7.4) | 22.8 (7.0) |  |
| [Range] | [0 to 32] | [0 to 32] |  |
| Centre for Epidemiologic Studies Depression scale (CES-D) |  |  | 0.0398 |
| Mean (SD) | 30.1 (12.3) | 31.2 (12.1) |  |
| [Range] | [0 to 59] | [1 to 60] |  |
| Short Form – 36 Physical Component Summary (PCS) |  |  | 0.0050 |
| Mean (SD) | 49.4 (11.0) | 50.7 (9.7) |  |
| [Range] | [12.2 to 67.6] | [19.7 to 69.3] |  |
| Short Form – 36 Mental Component Summary (MCS) |  |  | 0.0023 |
| Mean (SD) | 50.5 (9.7) | 49.3 (9.1) |  |
| [Range] | [29.3 to 83.9] | [30.9 to 77.1] |  |

^a^Separate logistic regression models for each variable

## Sensitivity analyses

### Completer-only analysis including participants returning all spin-17 outcome measures at all time points

To determine to what extent dilution of intervention effect due to non-compliance with returning outcomes was a factor in the primary analysis, a completer-only analysis was conducted. A completer was defined as a participant who returned all their outcome measures at baseline, 6 weeks, 3, 6 and 12 months. There was 310 (14.7%) completers; 70 (6.6%) completers in the E-Couch group and 240 (22.7%) completers in the control group. The summary statistics for the completer population is presented in e-Table 4 by randomized group at each time point.

e-Table 4 Descriptive summaries of mean and change in SPIN-17 score at each time point for each group for the completer population by randomized group

|  | E-Couch Social  Anxiety Module  N = 70 | Control  N = 240 |
| --- | --- | --- |
| Baseline |  |  |
| Mean (SD) | 35.23 (13.40) | 38.40 (13.84) |
| [Range] | [14 to 65] | [13 to 68] |
| 6 weeks |  |  |
| Mean (SD) | 28.34 (12.48) | 35.01 (14.37) |
| [Range] | [5 to 58] | [5 to 67] |
| Mean (SD) change from baseline  [Range] | -6.88 (9.98)  [-32 to 19] | -3.39 (9.15)  [-41 to 24] |
| 3 months |  |  |
| Mean (SD) | 26.11 (13.74) | 33.81 (14.44) |
| [Range] | [4 to 54] | [0 to 65] |
| Mean (SD) change from baseline  [Range] | -9.11 (11.78)  [-36 to 23] | -4.59 (9.73)  [-39 to 29] |
| 6 months |  |  |
| Mean (SD) | 24.41 (13.24) | 32.43 (14.89) |
| [Range] | [2 to 60] | [0 to 60] |
| Mean (SD) change from baseline  [Range] | -10.81 (13.08)  [-48 to 19] | -5.96 (11.20)  [-46 to 33] |
| 12 months |  |  |
| Mean (SD) | 23.14 (12.63) | 32.30 (15.43) |
| [Range] | [0 to 52] | [2 to 65] |
| Mean (SD) change from baseline  [Range] | -12.08 (13.81)  [-53 to 18] | -6.09 (12.02)  [-46 to 37] |

The adjusted mean treatment differences are presented in e-Table 5 .Model diagnostics were checked and residuals satisfied the normality assumption.

e-Table 5 Adjusted estimates from mixed effect model of change in SPIN-17 score at 6 weeks and 12 months for completers

|  | Adjusted difference in mean change [95% CI]^a^  P-value |
| --- | --- |
| E-Couch Social Anxiety Module vs Control (6 weeks) | -4.35 [-7.10 to -1.60]  P=0.0020 |
| E-Couch Social Anxiety Module vs Control (12 months) | -6.85 [-9.60 to -4.09]  P<0.0001 |

^a^Mixed effects linear model for change in SPIN-17 score from baseline, adjusted for baseline SPIN-17 score, time and interaction of time with randomized group and including a random effect for participants. Covariance matrix of within participant’s measurement was unstructured.

### Completer-only analysis including participants returning all spin-17 outcome measures at 6 weeks and 12 months only

A completer was defined as a participant who returned all their outcome measures at baseline, 6 weeks and 12 months. There were 860 (41%) completers; 246 (23.2%) completers in the E-Couch group and 614 (58.0%) completers in the control group. The summary statistics for the completer population is presented in e-Table 6 by randomized group at each time point.

e-Table 6 Descriptive summaries of mean and change in SPIN-17 score at each time point for each group for the completer population (6 weeks and 12 months) by randomized group

|  | E-Couch Social  Anxiety Module  N = 246 | Control  N = 614 |
| --- | --- | --- |
| Baseline |  |  |
| Mean (SD) | 37.62 (12.83) | 39.24 (13.71) |
| [Range] | [14 to 67] | [13 to 68] |
| 6 weeks |  |  |
| Mean (SD) | 31.54 (12.88) | 35.56 (14.13) |
| [Range] | [2 to 60] | [0 to 68] |
| Mean (SD) change from baseline  [Range] | -6.08 (10.19)  [-46 to 22] | -3.67 (9.21)  [-41 to 34] |
| 12 months |  |  |
| Mean (SD) | 27.32 (13.58) | 32.68 (14.99) |
| [Range] | [0 to 64] | [2 to 66] |
| Mean (SD) change from baseline  [Range] | -10.30 (12.99)  [-60 to 18] | -6.55 (11.36)  [-46 to 37] |

The adjusted mean treatment differences are presented in e-Table 7. Findings did not differ from the primary analysis. Model diagnostics were checked and residuals satisfied the normality assumption.

e-Table 7 Adjusted estimates from mixed effect model of change in SPIN-17 score at 6 weeks and 12 months for completers (at 6 weeks and 12 months only)

|  | Adjusted difference in mean change [95% CI]^a^  P-value |
| --- | --- |
| E-Couch Social Anxiety Module vs Control (6 weeks) | -2.83 [-4.34 to -1.33]  P<0.0001 |
| E-Couch Social Anxiety Module vs Control (12 months) | -4.17 [-5.67 to -2.67]  P<0.0001 |

^a^Mixed effects linear model for change in SPIN-17 score from baseline, adjusted for baseline SPIN-17 score, time and interaction of time with randomized group and including a random effect for participants. Covariance matrix of within participant’s measurement was unstructured.

### Primary analysis of SPIN-17 adjusting for covariates found to be associated with missing SPIN-17 at 6 weeks

The mixed model assumes that the data are missing at random (MAR). A logistic regression analysis was conducted to investigate factors that were predictive of non-response of the primary outcome, the results are presented in Table 3 Six baseline factors were identified as having an association with missing primary outcome; age (P<0.0001), marital status (P=0.0077), BFNE-S (P=0.0370), CES-D (P=0.0398), SF-36 PCS (0.0050) and SF36 MCS (P=0.0023).These were included as covariates in the model used in the primary analysis. Main conclusions did not differ from the primary analysis, see e-Table 8

e-Table 8 Adjusted estimates from mixed effect model of change in SPIN-17 at 6 weeks and 12 months Adjusted for covariates predicting missingness

|  | Adjusted difference in mean change [95% CI]^a^  P-value |
| --- | --- |
| E-Couch Social Anxiety Module vs Control (6 weeks) | -1.76 [-2.92 to -0.60]  P=0.0029 |
| E-Couch Social Anxiety Module vs Control (12 months) | -2.84 [-4.06 to -1.62]  P<0.0001 |

^a^Mixed effects linear model for change in SPIN-17 score from baseline, adjusted for baseline SPIN-17 score, time and interaction of time with randomized group, **baseline factors associated with missing of primary outcome (age, marital status, BFNE-S score, CES-D score, SF36 MCS score, SF36 PCS**) and including a random effect for participants. Covariance matrix of within participant’s measurement was unstructured.

### Missingness pattern of the primary outcome

The primary outcome, SPIN-17 score, is measured at 5 time points; baseline, 6 weeks, 3 months, 6 months, and 12 months follow-up. All participants have complete data for their SPIN-17 score at baseline. There are potentially 16 missing data patterns for SPIN-17 score at each of the 4 follow-up time points. The frequency and percentage of each missing data pattern for SPIN-17 score by randomized group is presented in e-Table 9

e-Table 9 Missingness pattern for the SPIN-17 scores at the follow-up time points by randomized group

| Missingness Pattern for SPIN-17 | Follow-Up Time Point  (🗸 = Observed) | | | | Randomized Group | | |
| --- | --- | --- | --- | --- | --- | --- | --- |
|  | **6 weeks** | **3 months** | **6 months** | **12 months** | **E-Couch Social Anxiety Module**  N= 1058 (%) | **Control**  N= 1058 (%) | **Total Randomized**  N = 2116(%) |
| All follow-up data is available | 🗸 | 🗸 | 🗸 | 🗸 | 70 (6.6) | 240 (22.7) | 310 (14.7) |
| Missing at 6 weeks |  | 🗸 | 🗸 | 🗸 | 11 (1.0) | 16 (1.5) | 27 (1.3) |
| Missing at 3 months | 🗸 |  | 🗸 | 🗸 | 96 (9.1) | 253 (23.9) | 349 (16.5) |
| Missing at 6 months | 🗸 | 🗸 |  | 🗸 | 31 (2.9) | 76 (7.2) | 107 (5.1) |
| Missing at 12 months | 🗸 | 🗸 | 🗸 |  | 10 (1.0) | 25 (2.4) | 35 (1.7) |
| Missing at 6 weeks and 3 months |  |  | 🗸 | 🗸 | 23 (2.2) | 34 (3.2) | 57 (2.7) |
| Missing at 6 weeks and 6 months |  | 🗸 |  | 🗸 | 14 (1.3) | 16 (1.5) | 30 (1.4) |
| Missing at 6 weeks and 12 months |  | 🗸 | 🗸 |  | 3 (0.3) | 6 (0.6) | 9 (0.4) |
| Missing at 3 and 6 months^a^ | 🗸 |  |  | 🗸 | 49 (4.6) | 45 (4.3) | 94 (4.4) |
| Missing at 3 and 12 months | 🗸 |  | 🗸 |  | 29 (2.7) | 30 (2.8) | 59 (2.8) |
| Missing at 6 and 12 months | 🗸 | 🗸 |  |  | 16 (1.5) | 38 (3.6) | 54 (2.6) |
| Missing at 3, 6 and 12 months | 🗸 |  |  |  | 114 (10.8) | 83 (7.8) | 197 (9.3) |
| Missing at 6 weeks, 6 and 12 months |  | 🗸 |  |  | 19 (1.8) | 11 (1.0) | 30 (1.4) |
| Missing at 6 weeks, 3 and 12 months |  |  | 🗸 |  | 23 (2.2) | 18 (1.7) | 41 (1.9) |
| Missing at 6 weeks, 3 and 6 months |  |  |  | 🗸 | 55 (5.2) | 30 (2.8) | 85 (4.0) |
| Missing at all follow-up time points |  |  |  |  | 495 (46.8) | 137 (13.0) | 632 (29.9) |

^a^There was an administrative error with the software sending automatic emails regarding questionnaire surveys which stopped working for about a month before it was spotted. Once this glitch was fixed the response period to have a ‘catch-up’ was extended from 4 weeks to 8 weeks for those participants who were missed but most were too late. As a consequence, follow-up data of outcome measures at 3 and 6 months data were very low.

A pattern mixture model was fitted to assess robustness of the Missing At Random (MAR) assumption required for the mixed effect regression model. Results are shown in Figure 1 indicating that even across a range of missing data patterns treatment difference would still be significant, indicating the robustness of the main results even with strong departures from MAR assumption.

e-Figure 1 Pattern mixture model results for SPIN-17 at 6 weeks

### Primary analysis assuming plausible arm specific differences (assuming data MNAR for SPIN-17 at 6 weeks)

Using the approach by White et al 2011 to carry out sensitivity analyses to investigate informative missing of SPIN-17 at 6 weeks, the following assumptions of differences between responders and non-responder were carried out:

- When the proportion of missing SPIN-17 at 6 weeks are assumed to the same in both arms (i.e. Both arms equally), assumes the mean of unobserved responses for SPIN-17 at 6 weeks could be as much as 75% more or 50% less (i.e. -50%) than the mean of observed responses
- When the data is assumed to be informatively missing only in the E-Couch arm (i.e. Only in E-Couch group), assumes the mean of unobserved responses for SPIN-17 at 6 weeks could be as much as 50% more or 50% less (i.e. -50%) than the mean of observed responses
- When the data is assumed to be informatively missing only in the Control arm (i.e. Only control group), assumes the mean of unobserved responses for SPIN-17 at 6 weeks could be as much as 50% more or 50% less (i.e. -50%) than the mean of observed responses
- Additionally, more moderate sensitivity analyses includes:
  - Data is informatively missing in both arms, assumes 50%*
  - Data is informatively missing in E-Couch arm assumed as much as 25% more*
  - Data is informatively missing in the Control arm assumed as much as 25% more*

e-Table 10 shows results when we assumed plausible arm specific differences of missing SPIN-17 score at 6 weeks between responders (defined has having SPIN-17 score and 6 weeks) and non-responders (missing SPIN-17 score at 6 weeks). The results indicates that even asymmetrical differences between responders and non-responders conclusions remain similar to the primary analysis, i.e. reduction in severity of social anxiety symptoms in people randomized to E-Couch compared to Control group.

e-Table 10 Sensitivity analysis using MNAR assumption for the effect of E-Couch on SPIN-17 at 6 weeks

| **Non-responders differ in** | **Assumed difference between non-responders and responders** | **Adjusted mean difference [95%CI]^b^** |
| --- | --- | --- |
| **Both arms equally** | -50 | -2.68 (-1.57 to -3.79) |
|  | 50^a^ | -2.29 (-3.40 to -1.18) |
|  | 75 | -2.24 (-3.35 to -1.13) |
| **Only E-Couch group** | -50 | -2.85 (-3.97 to -1.74) |
|  | 25^a^ | -2.30 (-3.41 to -1.19) |
|  | 50 | -2.20 (-3.30 to -1.07) |
| **Only Control group** | -50 | -2.26 (-3.37 to -1.15) |
|  | 25^a^ | -2.49 (-3.60 to -1.38) |
|  | 50 | -2.54 (-3.65 to -1.43) |

^a^Moderate sensitivity analysis, ^b^ adjusted for baseline SPIN-17 score

###

### System glitch at 6 months follow-up

There was an administrative error with the software sending automatic emails regarding questionnaire surveys which stopped working for about a month before it was spotted. Once this glitch was fixed the response period to have a ‘catch-up’ was extended from 4 weeks to 8 weeks for those participants who were missed but most were too late. As a consequence, follow-up data of outcome measures at 3 and 6 months data were very low.

The proportion of participants returning questionnaires at 6 months within 4 weeks split by randomized group are presented in e-Table 11.

e-Table 11 Proportion of participants returning 6 month follow-up questionnaires within 4 weeks by randomized group

| Time frame of completion of SPIN-17 at 6 months^a^ | E-Couch Social Anxiety Module  N = 265 (%) | Control  N = 622 (%) |
| --- | --- | --- |
| Completed in less than 4 weeks | 196 (74.0) | 452 (72.7) |
| Completed after 4 weeks | 69 (26.0) | 170 (27.3) |

**^a^**Time frame was calculated as follows, a date 6 months from randomization was calculated (used as date assuming when all participants would have received the 6 month questionnaire) and this date was used to calculate the timeframe gap (i.e. date submitted at 6 months – date 6 months from randomization)

## Subgroup analyses

Subgroup analyses was carried to assess which variables (pre-specified in the protocol) had an impact on the outcome.

### Subgroup analysis with change in SPIN-17 score at 6 weeks as outcome

Results shown in e-Figure 2 indicate that the intervention had a significantly greater impact on participants with high baseline SPIN-17 score in improvement of social anxiety symptoms (P=0.0135). No interaction effect was observed for other subgroups

e-Figure 2 Subgroup Forest plot for change in SPIN-17 at 6 weeks for E-Couch versus Control


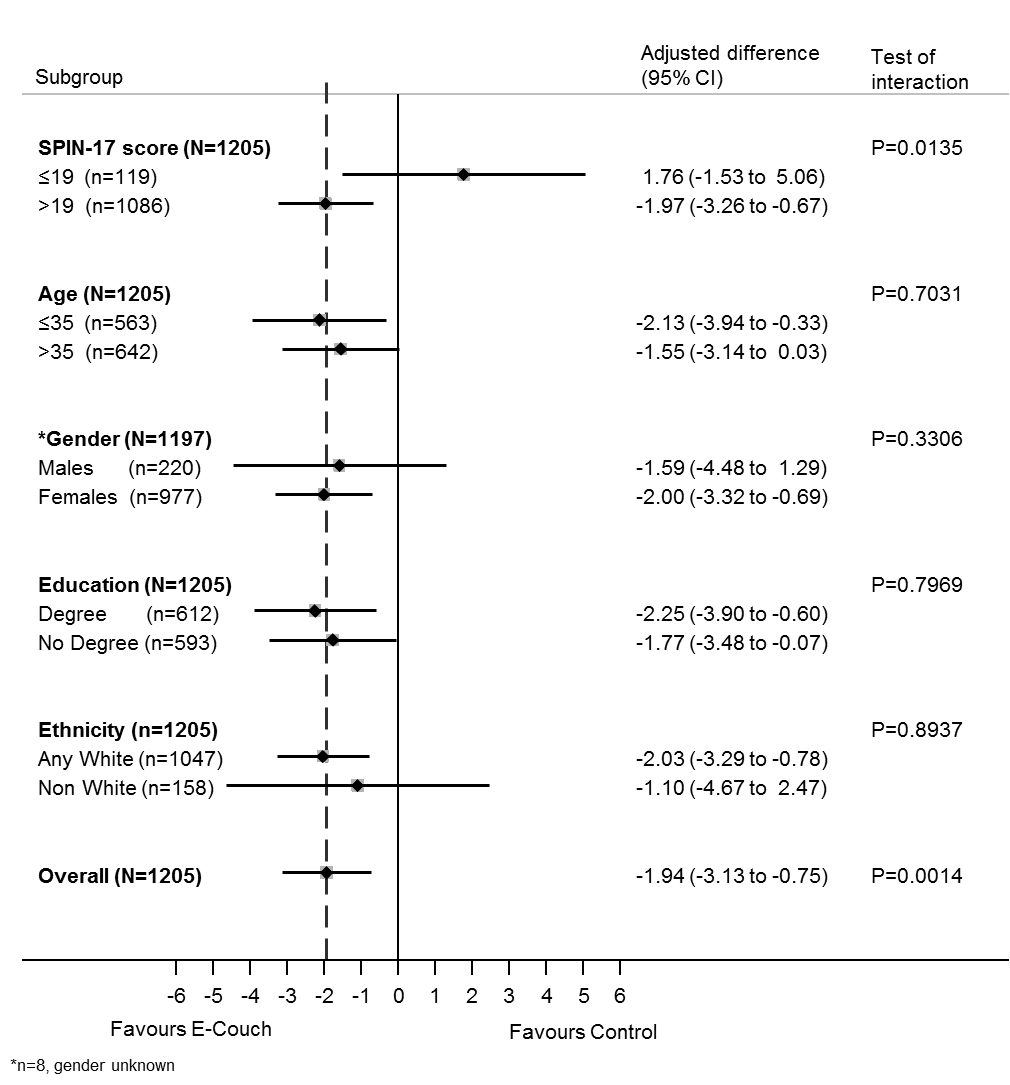


### Subgroup analysis with change in fear of negative evaluation (BFNE-S) at 6 weeks as outcome

Results shown in e-Figure 3 indicate there was no interaction effect in all subgroups considered.

e-Figure 3 Subgroup Forest plot for change in BFNE-S at 6 weeks for E-Couch versus Control


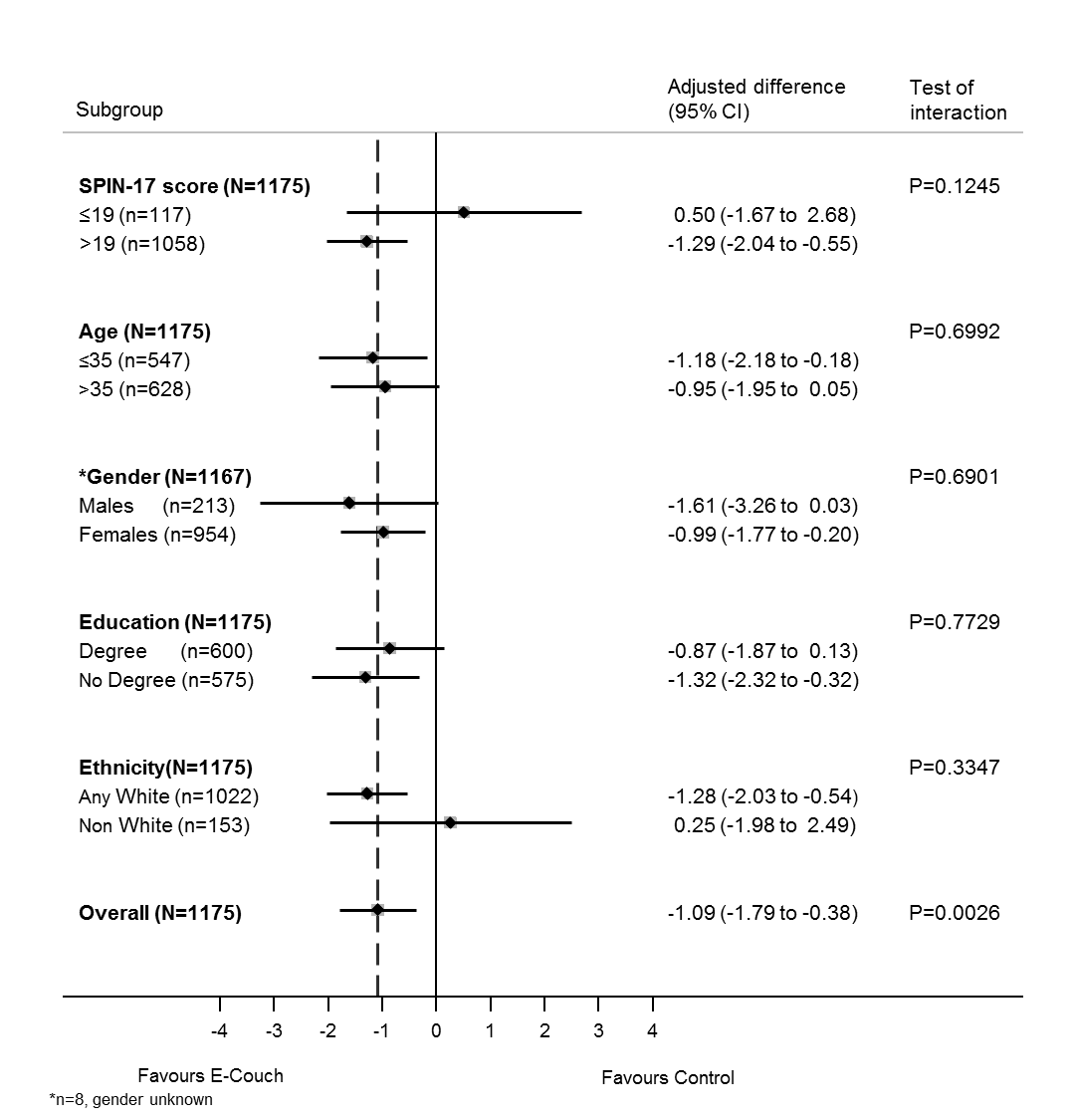


### Subgroup analysis with change in depression (CES-D) at 6 weeks as outcome

Results shown in e-Figure 4 indicate that the intervention had a significantly greater impact in participants with high baseline SPIN-17 score, (P=0.0070) and participants of white descent (P=0.0442). There was no interaction effect in all other subgroups.

e-Figure 4 Subgroup Forest plot for CES-D for E-Couch versus Control


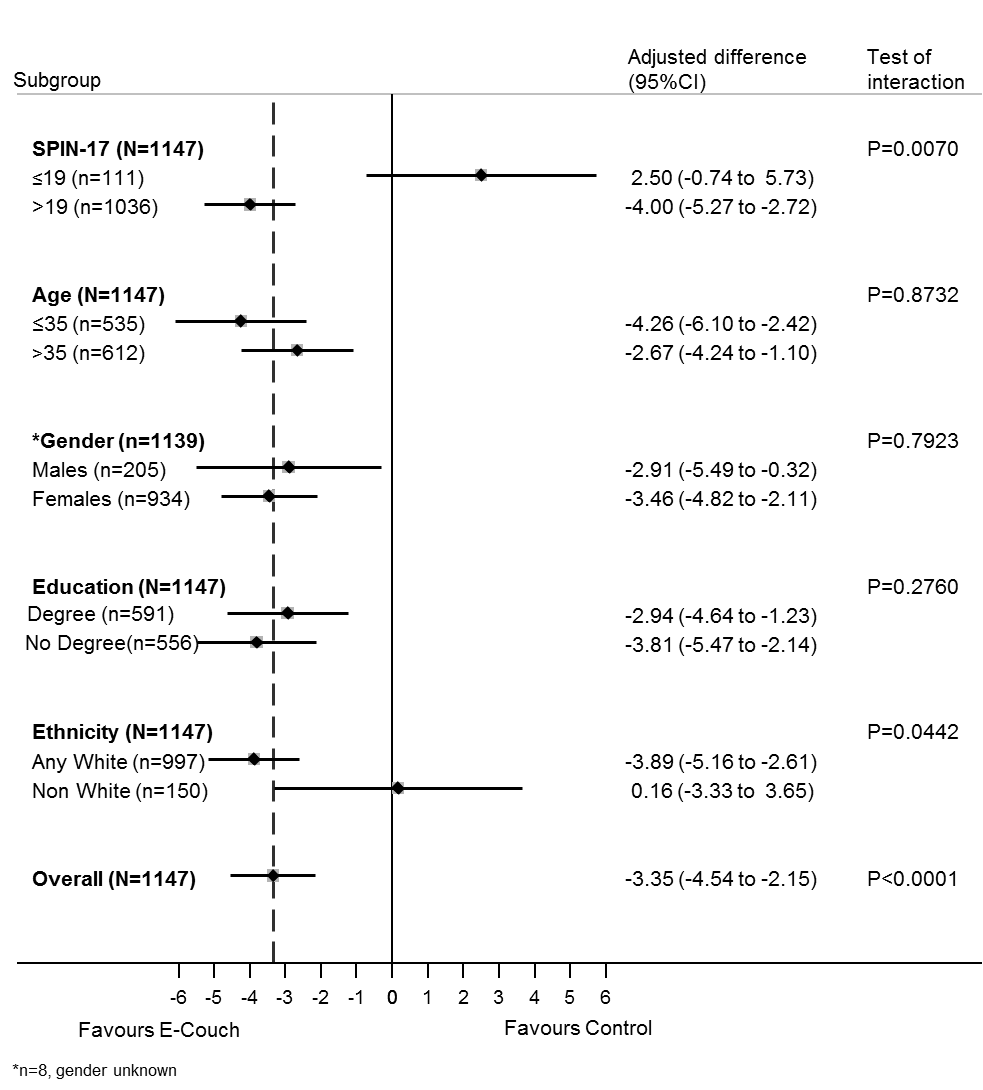


### Subgroup analysis with change in mental well-being (SWEMWBS) at 6 weeks as outcome

Results shown in e-Figure 5 there was no interaction effect in all subgroups considered.

e-Figure 5 Subgroup Forest plot for SWEMWBS for E-Couch versus Control


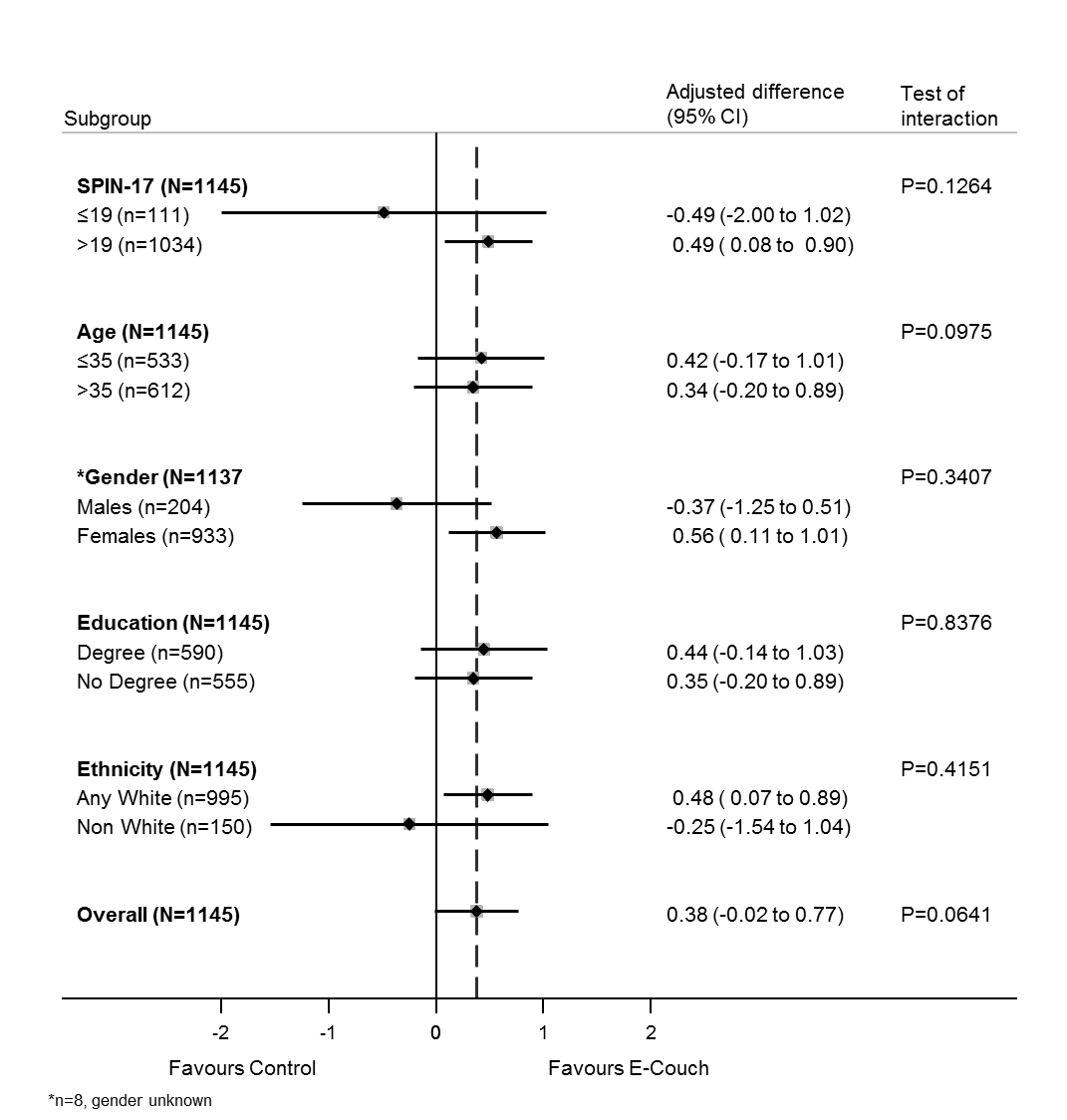


## Additional analysis

### Exploring difference in trend in SPIN-17 between randomized groups

An exploratory analysis was conducted to investigate change in SPIN-17 over time. The mixed effect model contained change in SPIN-17 score as the outcome and time as a continuous covariate to allow the slope of the regression line representing the change in outcome over time to be assessed between randomized groups. Even with time fitted as continuous, there was a significant difference between randomized groups, see e-Table 12

e-Table 12 Adjusted estimates from mixed effect model of change in SPIN-17 at 6 weeks with time fitted as a linear covariate in the model

|  | Adjusted mean difference [95% CI]^a^  P-value |
| --- | --- |
| E-Couch Social Anxiety Module vs Control | -1.60 [-2.98 to -0.23]  P=0.0220 |

^a^Mixed effects linear model of change in SPIN-17 score adjusting for baseline SPIN-17 score, time (fitted as continuous) and its interaction with randomized group and patient ID as a random effect

### Exploring adherence to E-couch social anxiety module

Adherence measured as number of modules completed over the study period. e-Table 13 shows the frequency of total number of modules completed in the E-Couch group median [IQR] was 1 [1 to 3].

e-Table 13 Summary of total modules completed over the study period

|  | E-Couch Social  Anxiety Module  N = 1058 (%) |
| --- | --- |
| Total number of modules completed |  |
| 1 | 428 (41) |
| 2 | 103 (10) |
| 3 | 55 (5) |
| 4 | 42 (4) |
| 5 | 57 (5) |
| 6 | 31 (3) |
| Did not start module | 342 (32) |
| Median modules completed [IQR] (n=716)  Mean modules completed (SD) [Range] (n=716) | 1 [1 to 3]  2 (1.52) [1 to 6] |

e-Table 14 Adjusted Poisson regression model for adherence in participants who received E-Couch intervention

|  | Estimate [95%CI]; P-value | Predicted number of modules complete |
| --- | --- | --- |
| Factors considered |  |  |
| Gender (Female vs Male) | 0.072 [-0.111 to 0.255]; P=0.4410 | 0.095 [-0.141 to 0.332]; P=0.4300 |
| Age (continuous) | 0.003 [-0.002 to 0.008]; P=0.3460 | 0.004 [-0.004 to 0.011]; P=0.3460 |
| Race (Any white vs other) | 0.177 [-0.213 to 0.566]; P=0.3740 | 0.221 [-0.228 to 0.670]; P=0.3340 |
| Baseline SPIN-17 score(continuous) | 0.000 [-0.005 to 0.005]; P=0.8980 | 0.000 [-0.007 to 0.008]; P=0.8980 |

None of the factors considered were found to be associated with adherence e-Table 14. For the purposes of interpretation, for example being female increases the expected log of modules completed by 0.072. The difference in expected counts of modules complete between females and males while holding all the other factors at their mean is 0.095 i.e. approx. 1.

### Description of Usage data, Adherence and Other help received (self-help and help from a health professional)

e-Table 15 shows the summary of usage data, adherence and other help received by randomized group. A higher proportion of participants randomized to the control group received help compared to the proportion of participants in the E-Couch group. Throughout the study follow-up, participants logged on to the site at least once and median time spent on the website was approx. 20 mins. With a median of approx. 30 page views and completing a median of 1 modules at each time point.

e-Table 15 Summary of usage data, adherence and other help received by randomized group

|  | E-Couch Social  Anxiety Module  (N = 1058) | Control  (N = 1058) |
| --- | --- | --- |
| Help from a health professional related to shyness or social anxiety |  |  |
| 6 weeks, n(%) | 54 (5) | 122 (12) |
|  |  |  |
| Self-help for problems related to shyness and social anxiety |  |  |
| 6 weeks, n(%) | 59 (6) | 167 (16) |
|  |  |  |
| Total number of logins to site |  |  |
| Median [IQR] |  |  |
| 6 weeks | 1 [0 to 2] | - |
| 3 months | 1 [0 to 3] | - |
| 6 months | 1 [0 to 3] | - |
| 12 months | 1 [0 to 3] | - |
| Total number of logins to site |  |  |
| Mean (SD) [Range] |  |  |
| 6 weeks | 1.67 (2.25) [0 to 19] | - |
| 3 months | 1.99 (2.84) [0 to 45] | - |
| 6 months | 2.14 (3.25) [0 to 56] | - |
| 12 months | 2.24 (3.52) [0 to 56] | - |
| Total time (minutes) spent on modules^a^ |  |  |
| Median [IQR] |  |  |
| 6 weeks | 19.3 [3.1 to 46.9] | - |
| 3 months | 21.2 [4.0 to 49.1] | - |
| 6 months | 21.5 [4.1 to 49.8] | - |
| 12 months | 21.6 [4.2 to 50.6] | - |
| Total time (minutes) spent on modules^a^ |  |  |
| Mean (SD) [Range] |  |  |
| 6 weeks | 35.3 (48.1) [0 to 369] | - |
| 3 months | 37.7 (51.3) [0 to 380] | - |
| 6 months | 38.6 (53.7) [0 to 499] | - |
| 12 months | 39.1 (54.1) [0 to 509] | - |
| Total page views |  |  |
| Median [IQR] |  |  |
| 6 weeks | 26 [9 to 50] | - |
| 3 months | 28 [10 to 56] | - |
| 6 months | 29 [10 to 57] | - |
| 12 months | 29 [11 to 59] | - |
| Total page views |  |  |
| Mean (SD) [Range] |  |  |
| 6 weeks | 37.6 (41.3) [0 to 358] | - |
| 3 months | 40.9 (44.7) [0 to 447] | - |
| 6 months | 42.0 (48.1) [0 to 543] | - |
| 12 months | 42.7 (48.7) [0 to 547] | - |
| Number of modules completed^b^ |  |  |
| Median [IQR] |  |  |
| 6 weeks (n=695) | 1 [1 to 2] | - |
| 3 months (n=710) | 1 [1 to 2] | - |
| 6 months (n=712) | 1 [1 to 3] | - |
| 12 months (n=716) | 1 [1 to 3] | - |
| Number of modules completed^b^ |  |  |
| Mean (SD) [Range] |  |  |
| 6 weeks (n=695) | 1.87 (1.43) [1 to 6] | - |
| 3 months (n=710) | 1.94 (1.48) [1 to 6] | - |
| 6 months (n=712) | 1.98 (1.50) [1 to 6] | - |
| 12 months (n=716) | 2.00 (1.52) [1 to 6] | - |

^a^Only time spent on modules was recorded. If the participant was inactive for more than 30 minutes, then participant was timed out from the website. For each content page view occurring within 30 minutes time between these page views were added to time on page count.

^b^Number of modules completed was checked between randomization date and each time point (e.g. module completion date was before 6 week from date of randomization, the ‘n’ included in each time point are shown)

### Dose-response effect of intervention

e-Table 16 and e-Table 17 shows change in SPIN-17 score at 6 weeks and 12 months respectively for usage data. Median change SPIN-17 at 6 weeks are presented as non-parametric tests were performed.

e-Table 16 Dose-response effect of E-Couch on change in SPIN-17 score at 6 weeks

|  | E-Couch Social Anxiety Module  (N = 415) | Change in SPIN-17 score at 6 weeks  Mean (SD) | Change in SPIN-17 score at 6 weeks  Median (IQR) | P-value^a^ |
| --- | --- | --- | --- | --- |
| Total number of modules |  |  |  | 0.0761 |
| (Modules not started) 0 | 83 | -4.55 (11.26) | -4.00 (-10 to 2) |  |
| 1 | 154 | -6.04 (9.92) | -6.00 (-12 to 0) |  |
| 2 | 57 | -6.09 (10.97) | -7.00 (-13 to 1) |  |
| 3 | 33 | -7.73 (9.42) | -8.00 (-15 to -3) |  |
| 4 | 30 | -7.63 (10.96) | -7.50 (-16 to 0) |  |
| 5 | 35 | -5.40 (13.23) | -4.00 (-13 to 4) |  |
| 6 | 23 | -10.65 (10.85) | -11.00 (-17 to -3) |  |
| Total number of logins to site (Median = 1) |  |  |  | 0.8251 |
| <1 | 78 | -5.95 (8.05) | -5.00 (-11 to -2) |  |
| ≥1 | 337 | -6.25 (11.30) | -6.00 (-13 to 1) |  |
| Total page views (Median = 26) |  |  |  | 0.0244 |
| <26 | 138 | -4.80 (10.26) | -4.00 (-10 to 1) |  |
| ≥26 | 277 | -6.90 (10.94) | -7.00 (-14 to 0) |  |
| Total time (minutes) spent on modules (Median = 19) |  |  |  | 0.0139 |
| <19 | 139 | -4.73 (10.54) | -4.00 (-9 to 0) |  |
| ≥19 | 276 | -6.94 (10.81) | -7.00 (-14 to 0) |  |

^b^Kruskall-Wallis test for medians

e-Table 17 Dose-response effect of E-Couch on change in SPIN-17 score at 12 months

|  | E-Couch Social Anxiety Module  (N = 349) | Change in SPIN-17 score at 12 months  Mean(SD) | Change in SPIN-17 score at 12 months  Median (IQR) | P-value^a^ |
| --- | --- | --- | --- | --- |
| Total number of accessed at 12 months |  |  |  | 0.2849 |
| (Modules not started) 0 | 50 | -7.25 (11.59) | -6.00 (-14 to -1) |  |
| 1 | 131 | -9.82 (11.79) | -10.00 (-16 to -2) |  |
| 2 | 46 | -9.43 (13.32) | -8.50 (-20 to -1) |  |
| 3 | 28 | -10.00 (10.90) | -10.00 (-16 to -3) |  |
| 4 | 33 | -12.15 (15.27) | -8.00 (-22 to 0) |  |
| 5 | 34 | -13.00 (16.33) | -9.50 (-25 to -2) |  |
| 6 | 27 | -12.89 (13.27) | -14.00 (-21 to -1) |  |
| Total number of logins to site (Median=1) |  |  |  | 0.3238 |
| <1 | 49 | -8.55 (12.08) | -8.00 (-14 to 0) |  |
| ≥1 | 300 | -10.31 (13.15) | -9.00 (-18 to -2) |  |
| Total page views (Median = 29) |  |  |  | 0.2843 |
| <29 | 100 | -8.93 (12.48) | -7.50 (-15 to -1) |  |
| ≥29 | 249 | -10.52 (13.20) | -10.00 (-19 to -2) |  |
| Total time spent on modules (Median = 22) |  |  |  | 0.0527 |
| <22 | 113 | -8.05 (13.09) | -7.00 (-15 to 0) |  |
| ≥22 | 236 | -11.02 (12.88) | -10.00 (-19 to -2) |  |

^a^Kruskall-Wallis test for medians

### Exploring if some modules are more beneficial than other in terms of change in SPIN-17 score

e-Table 18 shows the summary statistics for change in SPIN-17 at each time point for each module stratified by the status at which each participant got to with the module. Of the modules completed at 6 weeks, attention practice and exposure practice showed a higher positive change. This was also true at 12 months.

e-Table 18 Summary statistics of SPIN-17 score for each module by time point by status of module

|  | Status of Module accessed | | |
| --- | --- | --- | --- |
| Module name | **Not accessed**  **n, mean (SD)** | **Started**  **n, mean (SD)** | **Completed**  **n, mean (SD)** |
| Social anxiety information |  |  |  |
| 6 weeks (n=398) | 24, -3.71 (11.61) | 42 , -6.26 (12.71) | 332, -6.61 (10.61) |
| 3 months (n=173) | 6, 5.33 (15.78) | 19, -5.42 (7.57) | 148, -9.25 (11.56) |
| 6 months (n= 262) | 16, -3.94 (14.19) | 20, -10.05 (12.03) | 226, -9.37 (13.02) |
| 12 months (n = 348) | 21, -5.24 (12.87) | 28, -7.96 (11.46) | 299, -10.68 (13.04) |
| Exposure practice |  |  |  |
| 6 weeks (n=393) | 95, -6.23 (11.39) | 216, -5.89 (10.31) | 82, -8.07 (11.32) |
| 3 months (n=173) | 37, -3.76 (10.71) | 92, -8.52 (11.13) | 44, -11.75 (12.41) |
| 6 months (n= 261) | 51, -8.37 (13.82) | 158, -8.49 (12.59) | 52, -11.44 (13.62) |
| 12 months (n= 347) | 71, -8.24 (13.67) | 203, -10.17 (11.77) | 73, -11.89 (15.24) |
| Modify your thinking |  |  |  |
| 6 weeks (n=387) | 199, -5.96 (10.37) | 73, -6.34 (10.81) | 115, -7.26 (11.74) |
| 3 months (n=166) | 79, -6.49 (11.31) | 41, -9.59 (13.03) | 46, -10.17 (11.00) |
| 6 months (n= 261) | 121, -8.13 (12.08) | 48, -9.90 (11.45) | 92, -10.10 (14.85) |
| 12 months (n= 347) | 161, -8.86 (12.48) | 75, -10.97 (11.89) | 111, -11.14 (14.46) |
| Attention practice |  |  |  |
| 6 weeks (n=390) | 264, -5.53 (10.60) | 19, -4.74 (11.21) | 107, -8.33 (10.96) |
| 3 months (n=163) | 97, -6.56 (11.37) | 7, -12.29 (8.50) | 59, -10.32 (12.35) |
| 6 months (n= 261) | 159, -8.27 (12.27) | 15, -3.93 (14.99) | 87, -11.39 (13.86) |
| 12 months (n= 346) | 215, -8.62 (12.06) | 20, -6.40 (13.30) | 111, -13.21 (13.89) |
| Social skills training |  |  |  |
| 6 weeks (n=387) | 266, -5.37 (10.57) | 37, -9.62 (7.50) | 84, -7.25 (11.92) |
| 3 months (n=166) | 103, -7.14 (11.51) | 25, -12.24 (10.61) | 38, -9.21 (12.77) |
| 6 months (n= 261) | 160, -7.63 (12.66) | 28, -14.86 (11.64) | 73, -10.04 (13.76) |
| 12 months (n=347) | 219, -8.38 (12.23) | 38, -14.39 (13.60) | 90, -12.17 (14.00) |
| Relaxation |  |  |  |
| 6 weeks (n=385) | 270, -5.94 (10.43) | 35, -6.14 (11.41) | 80, -6.61 (11.64) |
| 3 months (n=164) | 103, -7.72 (10.91) | 19, -10.63 (12.14) | 42, -8.00 (12.61) |
| 6 months (n=257) | 164, -8.11 (12.14) | 21, -15.33 (14.35) | 72, -10.82 (13.47) |
| 12 months (n= 346) | 222, -8.98 (12.05) | 37, -12.70 (14.74) | 87, -11.95 (14.09) |

###

### Mediation analysis

Mediation analysis was done to

- To find out if more interaction with the website (i.e. total time spent on site and number logins) had an impact on higher levels of treatment effect
- To find out if receiving help for sub clinical social anxiety symptoms during the trial also had a higher impact on treatment effect
- To find out if compliance (i.e. modules completed) had an influence on treatment effect

e-Table 19 shows mediation analysis results when considering mediators at 6 weeks with change in SPIN-17 at 12 months as the outcome. There was no evidence of a direct effect of E-Couch on SPIN-17 mean (SE) -1.52 (1.00); P=0.1300. The estimated mediated effect of E-Couch on change in social anxiety symptoms was an improvement in change of SPIN-17 score of -1.58 (0.61); P=0.0108. The proportion of the effect of E-Couch on SPIN-17 that was mediated by total number of logins is 0.51. All other factors considered did not mediate SPIN-17 score at 12 months.

e-Table 19 Estimates of indirect effect for mediators at 6 weeks for change in SPIN-17 outcome at 12 months from mediation analysis

| **Mediator** | **Tested Pathway** | **Effect estimate** | **Effect size (SE); P-value** |
| --- | --- | --- | --- |
| Total logins at 6 weeks | E-couch SPIN | Total effect c | -3.09 (0.79); P <0.0001 |
|  | E-couch SPIN \| *Total logins* | Direct effect c’ | -1.52 (1.00); P=0.1300 |
|  | E-couch *Total logins* SPIN | Indirect effect | -1.58 (0.61); P=0.0108 |
|  |  | Proportion mediated  [Indirect/total effect] | 0.51 |
|  |  |  |  |
|  |  |  |  |
| Total time (minutes) spent on site at 6 weeks | E-couch SPIN | Total effect c | -3.09 (0.79); P <0.0001 |
|  | E-couch SPIN \| *Total time* | Direct effect c’ | -2.47 (1.00); P=0.0124 |
|  | E-couch *Total time* SPIN | Indirect effect | -0.63 (0.59); P=0.2897 |
|  |  | Proportion mediated  [Indirect/total effect] | 0.20 |
|  |  |  |  |
|  |  |  |  |
| Number of modules completed at 6 weeks | E-couch SPIN | Total effect c | -3.09 (0.79); P<0.0001 |
|  | E-couch SPIN \| *Modules* | Direct effect c’ | -2.10 (0.96); P=0.0287 |
|  | E-couch *Modules* SPIN | Indirect effect | -1.00 (0.54); P=0.0660 |
|  |  | Proportion mediated  [Indirect/total effect] | 0.32 |
|  |  |  |  |
|  |  |  |  |
| Other help received at 6 weeks^a^ | E-couch SPIN | Total effect c | -0.12 (0.03); P<0.0001 |
|  | E-couch SPIN \| *Other help* | Direct effect c’ | -0.12 (0.03); P<0.0001 |
|  | E-couch *Other help* SPIN | Indirect effect^b^ | 0.0003 (0.001); P=0.8400 |
|  |  | Proportion mediated  [Indirect/total effect] | 0.0025 |
|  |  |  |  |
|  |  |  |  |

^a^ Logistic regression used for mediator. ^b^Used bootstrap estimations to obtain SE’s
