# Supplementary material for: Effectiveness and Cost-Effectiveness of a Self-Guided Internet Intervention for Social Anxiety Symptoms in a General Population Sample: Randomized Controlled Trial
Source: J Med Internet Res. 2020 Jan 10;22(1):e16804. doi: 10.2196/16804 (PMC6996778; doi:10.2196/16804)
Supplement: Multimedia Appendix 3 [file jmir_v22i1e16804_app3.docx]

Descriptive statistics of Social Phobia Inventory (SPIN-17) score at all time points by randomized group.

| Time point | | SPIN-17^a^ | | BFNE-S^b^ | | CES-D^c^ | | SWEMWBS^d^ | | SF-36 (PCS)^e^ | | SF-36 (MCS)^f^ | |
| --- | --- | --- | --- | --- | --- | --- | --- | --- | --- | --- | --- | --- | --- |
|  | | E-Couch (N=1058) | Control (N=1058) | E-Couch (N=1058) | Control (N=1058) | E-Couch (N=1058) | Control (N=1058) | E-Couch (N=1058) | Control (N=1058) | E-Couch (N=1058) | Control (N=1058) | E-Couch (N=1058) | Control (N=1058) |
| **Baseline** | | | | | | | | | | | | | |
|  | n | 1058 | 1058 | 1058 | 1058 | 1058 | 1058 | 1058 | 1058 | 1058 | 1058 | 1058 | 1058 |
|  | Mean (SD) | 39.61 (13.14) | 39.83 (13.43) | 22.49 (7.11) | 22.4 (7.32) | 30.50 (12.26) | 30.72 (12.19) | 17.71 (3.10) | 17.73 (3.32) | 50.17 (10.29) | 49.81 (10.60) | 49.94 (9.48) | 50.06 (9.44) |
|  | Range | 13 to 68 | 13 to 68 | 0 to 32 | 0 to 32 | 2 to 60 | 0 to 58 | 7 to 31 | 7 to 35 | 12.2 to 69.3 | 16.8 to 67.3 | 29.3 to 77.1 | 30.9 to 83.9 |
| **6 weeks** | | | | | | | | | | | | | |
|  | n | 415 | 790 | 397 | 778 | 385 | 762 | 384 | 761 | 376 | 752 | 376 | 752 |
|  | Mean (SD) | 32.57 (13.46) | 35.78 (14.31) | 20.24 (7.96) | 21.69 (8.21) | 23.50 (9.35) | 28.27 (13.23) | 18.88 (3.89) | 18.26 (3.86) | 49.73 (10.69) | 49.73 (10.70) | 51.19 (9.33) | 49.38 (9.48) |
|  | Range | 2 to 65 | 0 to 68 | 1 to 32 | 0 to 32 | 7 to 54 | 1 to 60 | 7 to 35 | 7 to 35 | 15.9 to 70.2 | 16.4 to 68.5 | 30.7 to 78.3 | 21.1 to 73.8 |
|  | Mean (SD) change from baseline, range | −6.20 (10.76), −48 to 23 | −3.99 (9.28), −46 to 34 | −1.79 (6.12), −24 to 17 | −0.50 (5.10), −32 to 27 | −5.58 (9.77), −36 to 21 | −2.22 (9.45), −41 to 29 | 0.95 (3.15), −8.8 to 16.4 | 0.49 (2.84), −9.2 to 14.3 | 0.84 (6.44), −21.0 to 21.1 | 0.40 (6.23), −27.8 to 23.7 | −0.16 (8.42), −25.9 to 29.6 | −0.97 (7.54), −28.7 to 23.3 |
| **3 months^b^** | | | | | | | | | | | | | |
|  | n | 174 | 428 | 171 | 419 | 168 | 414 | 167 | 413 | 166 | 402 | 166 | 402 |
|  | Mean (SD) | 30.15 (14.82) | 34.31 (14.41) | 18.65 (8.77) | 21.54 (8.39) | 23.53 (13.86) | 27.00 (12.73) | 19.42 (4.41) | 18.32 (3.92) | 49.83 (9.75) | 50.06 (10.58) | 52.47 (9.60) | 48.94 (9.22) |
|  | Range | 4 to 65 | 0 to 68 | 0 to 32 | 0 to 32 | 2 to 59] | 0 to 58 | 7 to 33] | 7 to 35 | 21.8 to 68.2] | 19.2 to 66.8 | 27.6 to 73.6 | 27.5 to 72.8 |
|  | Mean (SD) change from baseline, range | −8.20 (11.73), −45 to 23 | −5.16 (9.72), −46 to 29 | −3.01 (6.51), -20 to 15 | −0.86 (5.45), −31 to 19 | −5.23 (11.41), −38 to 31 | −3.06 (10.15), −40 to 23 | 1.35 (3.69), −14.5 to 14.6 | 0.47 (3.40), −28.0 to 19.0 | 0.02 (7.21), −28.1 to 21.0 | −0.40 (7.34), −25.8 to 23.5 | 0.70 (9.10), −28.1 to 28.6 | −1.38 (8.60), −28.4 to 25.1 |
| **6 months^g^** | | | | | | | | | | | | | |
|  | n | 265 | 622 | 260 | 607 | 252 | 598 | 252 | 596 | 249 | 590 | 249 | 590 |
|  | Mean (SD) | 28.66 (14.38) | 33.26 (14.74) | 17.69 (8.69) | 20.81 (8.89) | 23.58 (13.38) | 26.62 (13.71) | 19.68 (4.24) | 18.65 (4.08) | 49.36 (10.80) | 50.23 (10.38) | 52.04 (9.48) | 49.12 (9.28) |
|  | Range | 2 to 68 | 0 to 68 | 0 to 32 | 0 to 32 | 0 to 57 | 0 to 60 | 7 to 35 | 7 to 35 | 17.7 to 66.8 | 19.9 to 67.2 | 30.4 to 71.6 | 26.2 to 74.4 |
|  | Mean (SD) change from baseline, range | −8.92 (13.16), −53 to 40 | −5.70 (10.59), -46 to 39 | −4.04 (7.36), -27 to 19 | −1.00 (5.85), -26 to 22 | −5.21 (11.36), -38 to 27 | −3.20 (10.86), -43 to 27 | 1.67 (3.68), −9.9 to 14.3 | 0.75 (3.60), −25.6 to 15.4 | 1.60 (6.82), −17.7 to 24.6 | 1.02 (7.47), −28.0 to 33.8 | 0.01 (9.23), −23.6 to 30.2 | −1.91 (8.76), −29.9 to 26.7 |
| **12 months** | | | | | | | | | | | | | |
|  | n | 349 | 710 | 335 | 699 | 328 | 690 | 327 | 683 | 322 | 674 | 322 | 674 |
|  | Mean (SD) | 27.92 (14.09) | 32.35 (15.11) | 17.67 (9.23) | 20.54 (9.01) | 22.58 (13.62) | 25.72 (13.22) | 19.95 (4.35) | 18.97 (4.22) | 50.24 (10.03) | 49.88 (10.67) | 51.72 (9.59) | 49.16 [9.21] |
|  | Range | 0 to 67 | 0 to 66 | 0 to 32 | 0 to 32 | 0 to 60 | 0 to 60 | 7 to 35 | 7 to 35 | 17.8 to 70.0 | 13.7 to 69.3 | 25.1 to 70.4 | 27.0 to 73.0 |
|  | Mean (SD) change from baseline, range | −10.06 (13.00), −60 to 25 | −6.97 (11.65), −47 to 37 | −4.06 (7.97), −30 to 22 | −1.52 (6.53), −29 to 21 | −6.22 (12.46), −47 to 36 | −4.39 (11.56), −46 to 42 | 2.05 (4.03), −8.8 to 20.3 | 1.11 (3.64), −9.9 to 17.6 | 0.46 (7.70), −23.6 to 33.7 | 0.37 (8.27), −32.5 to 35.3 | 0.65 (9.80), −32.8 to 31.8 | −1.51 (9.55), −33.4 to 31.0 |

^a^Primary outcome.

^b^BFNE-S: Brief Fear of Negative Evaluation score.

^c^CES-D: Centre for Epidemiologic Studies Depression scale.

^d^SWEMWBS: Short Warwick-Edinburgh Mental Well-Being Scale.

^e^SF-36 (PCS): Short Form-36 (physical component summary).

^f^SF-36 (MCS): Short Form-36 (mental component summary).

^g^There was an administrative error with the software sending automatic emails regarding questionnaire surveys which stopped working for about a month before it was spotted. Once this glitch was fixed, the response period to have a “catch-up” was extended from 4 weeks to 8 weeks for those participants who were missed but most were too late. As a consequence, follow-up data of outcome measures at 3 and 6 months were very low.
